# Modeling Plasma-Induced Modifications in Alginate Biopolymers at the Atomic Scale

**DOI:** 10.1021/acs.jpcc.5c01565

**Published:** 2025-05-01

**Authors:** Maksudbek Yusupov, Francesco Tampieri, Shakhrizoda Matnazarova, Nosir Matyakubov, Cristina Canal, Annemie Bogaerts

**Affiliations:** † Institute of Fundamental and Applied Research, National Research University TIIAME, Tashkent 100000, Uzbekistan; ‡ Department of Information Technologies, Tashkent International University of Education, Tashkent 100207, Uzbekistan; § Research Group PLASMANT, Department of Chemistry, University of Antwerp, Antwerp 2610, Belgium; ∥ Biomaterials, Biomechanics and Tissue Engineering Group, Department of Materials Science and Engineering and Institute for Research and Innovation in Health (IRIS), Universitat Politècnica de Catalunya—BarcelonaTech (UPC), Barcelona 08019, Spain; ⊥ Barcelona Research Centre in Multiscale Science and Engineering (CCEM), UPC, Barcelona 08019, Spain; # Centro de Investigación Biomédica en Red de Bioingeniería, Biomateriales y Nanomedicina (CIBER-BBN), Instituto de Salud Carlos III, Madrid 28029, Spain; ∇ Arifov Institute of Ion-Plasma and Laser Technologies, Academy of Sciences of Uzbekistan, Tashkent 100125, Uzbekistan; ○ Department of Physics, Urgench State University, Urgench 220100, Uzbekistan

## Abstract

This study investigates the impact of reactive oxygen species produced by nonthermal plasma on biopolymers using computer simulations. Specifically, reactive molecular dynamics simulations are employed to study the interaction between oxygen atoms—a key short-lived component generated during direct plasma treatment—and the alginate molecule, which serves as the model system in our analysis. The simulations reveal that oxygen atom impact leads to significant structural changes, including oxygen addition (44.5%), glycosidic bond cleavage (13.5%), ring opening (31%), and organic peroxide formation (25%) (these events are not mutually exclusive, and therefore, the percentages do not sum directly to 100%). Additionally, the oxidation process results in carboxyl group reduction and CO_2_ detachment (13%), potentially altering the cross-linking properties of alginate. Our model results align with existing experiment findings and provide deep insight into the interaction between alginate and plasma-generated reactive species. This is fundamental for the use of biopolymers, particularly those capable of forming hydrogels, combined with plasma, for biomedical applications.

## Graphical abstract

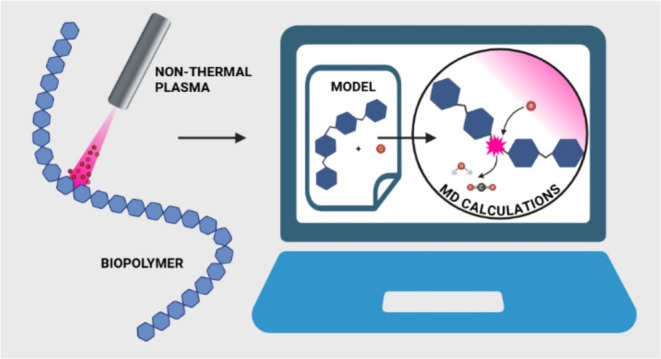

## Introduction

Plasma medicine is an emerging interdisciplinary field that utilizes nonthermal plasma (NTP) for various medical applications, including wound healing, cancer treatment, and sterilization. Through the generation of reactive oxygen and nitrogen species (RONS), NTP can selectively target pathogens and cancer cells, while promoting tissue regeneration. This noninvasive approach holds great promise for combating antibiotic resistance and enhancing chronic wound care.^1^

Plasma-generated RONS can be delivered to tissues either directly, using a plasma source, or indirectly via a carrier medium, such as gas or liquid. During direct plasma treatment, all components of CAP (free electrons, ions, radicals, excited species, neutral species and electromagnetic radiation) can interact with the target, while in indirect treatments, only the long-lived chemical species survive.^2^ When the liquids that are treated by CAP contain organic molecules, several possible products can be generated that may have important biological effects.^3,4^ Recently, hydrogels composed of natural or synthetic polymers have been explored as carriers for plasma-generated RONS. Unlike liquids that are quickly diluted by body fluids, hydrogels can retain reactive species for extended periods of time, enhancing their therapeutic potential.^4–6^

The primary hydrogels investigated for applications in plasma medicine are mainly composed of natural polysaccharides, with alginate, agarose, and cellulose derivatives being the most studied.^7^ Recent studies have demonstrated that directly treating these biopolymers with NTP before cross-linking enables efficient storage and delivery of RONS. Additionally, NTP treatment has been shown to alter the structure of these biopolymers, leading to changes in viscosity, molecular weight distribution, and mechanical properties.^6,8^

While numerous studies have examined the oxidative effects of NTP on biopolymers, the underlying molecular mechanisms remain complex due to the intricate interactions between reactive species and biopolymer structures. A deeper understanding of these interactions, particularly in polysaccharides like alginate, is crucial for optimizing their plasma-based biomedical applications.

There are several experimental investigations in the literature devoted to the effects of NTP on polysaccharides. For instance, it was demonstrated that mono- and polysaccharides undergo chemical modifications when exposed to RF-plasma, resulting in an average formation of one carbonyl group per monosaccharide unit on both the surface and bulk of the NTP-treated monosaccharides.^9^ It was also found that NTP treatment of an aqueous glucose solution primarily produces acids, such as oxalic, glycolic, tartaric, glyceric, and formic acid, linked to reactive oxygen species (ROS), particularly hydroxyl radicals.^10^ Similarly, a recent study showed that short-lived species like hydroxyl radicals (OH) and particularly atomic oxygen (O­(^3^P) radicals) play a significant role in the glucose oxidation cascade, while long-lived hydrogen peroxide (H_2_O_2_) is merely a byproduct and not a key oxidant.^11^ It was suggested that O atoms are the primary oxidant in Ar/O_2_ admixture plasma with or without N_2_ shielding flow,^11^ capable of directly oxidizing organic molecules in aqueous solutions without intermediates.^12^ In,^13^ it was reported that NTP treatment decreases oligosaccharide chain lengths by four to seven glycosidic-linked saccharide monomers, similar to its effects on fructose, likely due to the reaction with plasma-generated reactive species. It was also observed that NTP-induced reactive species interact with cellulose, cleaving glycosidic bonds and releasing short-chain cellodextrins that form branched glucans.^14^ Nevertheless, the aforementioned experimental findings provide limited insights into the molecular-level effects of NTP on saccharides.

Computer simulations can complement experiments by revealing atomic-level processes. In the literature, several atomistic simulation studies have explored the interactions of plasma-generated species with biomolecules containing disaccharides. Our previous molecular dynamics (MD) research showed that ROS interactions (e.g., O atoms and OH radicals) lead to glycosidic bond cleavage and ring opening in the disaccharides of peptidoglycan, a component of Gram-positive bacterial cell wall.^15,16^ Similar outcomes were noted in lipid A, a constituent of Gram-negative bacteria, where interactions with ROS resulted in the dissociation and formation of crucial bonds, leading to the cleavage of glycosidic bonds and the opening of sugar rings.^17,18^ It was also found that OH radicals initiate reactions in *N*-acetylglucosamine through H-abstraction, resulting in ring opening and molecular damage.^19^ Similarly, it was reported that ROS, particularly O and OH species, have the capability to break C–C and C–O bonds in β1,6-glucan, leading to bond dissociation and ring opening in sugar monomers.^20^ Further studies with β-glucan and chitin polymers also confirmed that O and OH species readily break the glycosidic bonds of these oligosaccharides.^21,22^ Research on poly-β-1–6-*N*-acetylglucosamine showed that OH radicals cause not only H-abstraction, thereby destroying the polysaccharide, but also OH addition to the sugar structure.^23^ Yin et al. noted that ROS interactions with cellulose polysaccharide lead to aldehyde and vinyl group formation and reduce OH groups and pyran rings.^24^ While the above computational studies concentrate on the collective impacts of ROS, understanding the effects of individual ROS, such as O atoms, is crucial. Recent research has revealed that O atoms cause OH addition and ring opening in hyaluronan oligosaccharides,^25^ potentially leading to degradation and reduced molecular weight.^26,27^ Similarly, in our recent studies on cellotriose and glucuronic acid, we observed the effect of oxidative modification and degradation caused by O atoms, which is consistent with our experimental data on the effect of NTP on these oligosaccharides.^8,28^

The aim of this work is to assess, through *in silico* methods, the potential modifications to the structure of a polysaccharide like alginate, induced by short-lived ROS generated by NTP, and to validate these results by comparing them with existing experimental data. Thus, in this study, we examine the influence of a single ROS impact on an alginate tetramer molecule, serving as a simple model system for the biopolymer. In particular, we use reactive MD simulations to study the nonconsecutive impact of O atoms, a crucial ROS component (representing the behavior of other ROS^15–17,25,28^ ), on an intact alginate molecule. The novelty of this study compared to our previous work on the glucuronic acid monomer^8^ is the use of a more realistic model, the tetramer, which allows us to investigate NTP effects on all possible (repeating) bonds in the alginate polysaccharide, including the glycosidic bonds that are absent in the monomer model and are critical for the structural integrity, stability and function of biopolymers.

## Computational Details

To gain a deeper understanding of the effects of oxygen plasma on biopolymers like alginate and to provide atomistic insights into the reaction mechanisms, we conducted reactive MD simulations using the density functional-tight binding (DFTB) potential (DFTB-MD).^29^ Specifically, we employed the DFTB3 method, an extended version of self-consistent charge DFTB.^30^ To describe interatomic interactions in our simulations, we used the “3ob-3–1” parameter set adapted for DFTB3 and suitable for organic and biomolecules.^31,32^ Note that the DFTB method was selected over classical reactive force field approaches (e.g., ReaxFF) because it offers a more accurate description of electronic effects such as bond polarization, charge transfer, and radical formation, which are critical in oxidation reactions. Although ReaxFF simulations are computationally less demanding and allow for larger system sizes, they rely on empirical parameterizations and may not capture subtle electronic effects with sufficient accuracy, particularly in reactions involving highly reactive species such as atomic oxygen. DFTB thus provides a better balance between computational cost and electronic structure accuracy, making it more appropriate for modeling plasma-induced chemical modifications at the atomic scale.

As a model system, we used the tetramer structure of alginic acid (i.e., the protonated form of alginate) consisting of two guluronic acid and two mannuronic acid residues connected by β­(1,4) glycosidic bonds (Figure 1). This model system includes all possible bonds that are repeated in the alginate polysaccharide, which allows us to study these bonds, namely their dissociation and/or the formation of new bonds that occur in the alginate molecule when it interacts with an O atom, one of the reactive species generated by NTP.^33^

**Figure 1:**
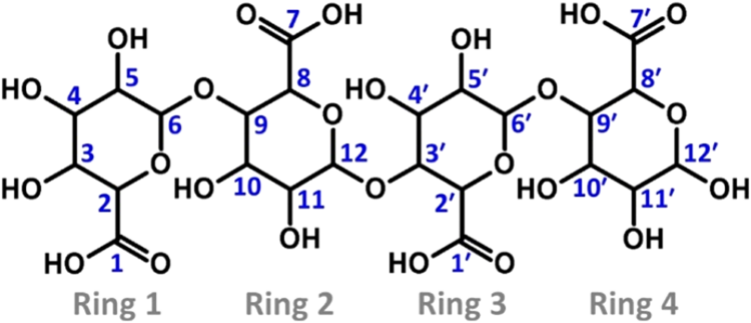
Chemical structure of the alginic acid (or alginate) tetramer comprising two guluronic acid units (rings 1 and 2) and two mannuronic acid units (rings 3 and 4). For clarity, H atoms bound to C atoms are not shown. This convention is also followed in subsequent similar figures. All C atoms are numbered in the structure, which might participate in the interaction of O atoms with the system.

The model system (i.e., alginic acid tetramer, C_24_H_34_O_25_, 83 atoms, 722 g/mol) in our simulations was prepared as follows. Initially, the tetramer structure was placed in a 40 Å × 40 Å × 40 Å simulation box with periodic boundary conditions applied in all three directions. This box size was sufficient to randomly generate a single O atom around the structure (see below). The model system was then energy minimized using the conjugate gradient algorithm, followed by equilibration (thermalization) for 1200 ps in a canonical NVT ensemble at 300 K, employing the Berendsen thermostat^34^ with a coupling constant of 100 fs. This equilibration time was sufficient for obtaining a well-thermalized structure; see Figure S1 in the Supporting Information (SI). Subsequently, a single O atom was randomly generated around the structure, maintaining a minimum distance of 7 Å from the molecule, i.e., a distance larger than the cutoff radius used for nonbonded interactions. This ensured a homogeneous and unbiased spatial distribution of impacts, while avoiding any initial long-range or short-range interactions. Under these conditions, 200 impact simulations (i.e., 200 DFTB-MD runs) were carried out for a total simulation time of 200 ps (i.e., 4 × 10^5^ iterations) per simulation, which was sufficient to observe breaking and formation of bonds in the structure. While further stabilization of reaction products could occur on longer time scales or require overcoming high activation barriers, the present study focused on capturing the initial oxidative events following O atom impacts. In all simulations (i.e., during the thermalization, as well as during the particle impact simulations) a time step of 0.5 fs was used. All simulations were conducted using the DFTB+ package, version 19.1.^35^

As mentioned above, we conducted 200 DFTB-MD simulations to comprehensively study the mechanisms of all potential bond-breaking and formation processes following a random impact of an O atom on the structure, as well as to gather some (limited) statistics on the observed mechanisms. It is noteworthy that we did not include an aqueous layer covering the model molecule due to the high computational cost associated with the DFTB method, as simulating the system with a water layer would require excessive calculation time, especially when aiming for statistically meaningful sampling. Although using a faster (but less accurate than DFTB) reactive force field such as ReaxFF could make simulations with explicit water more tractable, performing a large number of trajectories (i.e., 200 MD runs) would still be highly time-consuming. On another hand, our study focused exclusively on the impact of an O atom and its nonconsecutive interaction to determine the individual ROS (i.e., O atom) effects on alginate oxidation and to compare the simulation results with experimental data. It should be noted that O atoms can react with water molecules to generate OH radicals during plasma-liquid interactions. Our previous simulation studies demonstrated that O atoms and OH radicals trigger very similar reaction mechanisms in saccharides and polysaccharides, with O atoms effectively behaving as two OH radicals.^15–17,25,28^ Based on this mechanistic similarity, it is justified to focus only on O atom impacts to represent the primary oxidative modifications. Indeed, a similar modeling approach was adopted in our recent studies on cellotriose and glucuronic acid molecules, where simulations of O atom interactions provided results largely consistent with experimental mass spectrometry findings on plasma-treated saccharides.^8,28^ For clarity, from this point onward, we refer to our model system as alginate in general, as it is more relevant for biological applications.

## Results

Our reactive MD simulations reveal the effects of nonconsecutive impacts of O atoms on an intact alginate tetramer. In total, out of 200 runs, we identified 85 reaction mechanisms, detailed in Table S1 of the SI. Here, we focus on the main results, which are also summarized in Figure 2. It should be noted that the reported probabilities correspond to the fraction of simulations where each specific outcome was observed, regardless of co-occurrence with other outcomes; consequently, the sum of the percentages exceeds 100%. This is exemplified by mechanism No. 71 (Table S1, SI), where multiple reaction outcomes occur simultaneously, including aldehyde group formation, ring opening, glycosidic bond cleavage, and liberation of CO_2_ and H_2_O.

**Figure 2:**
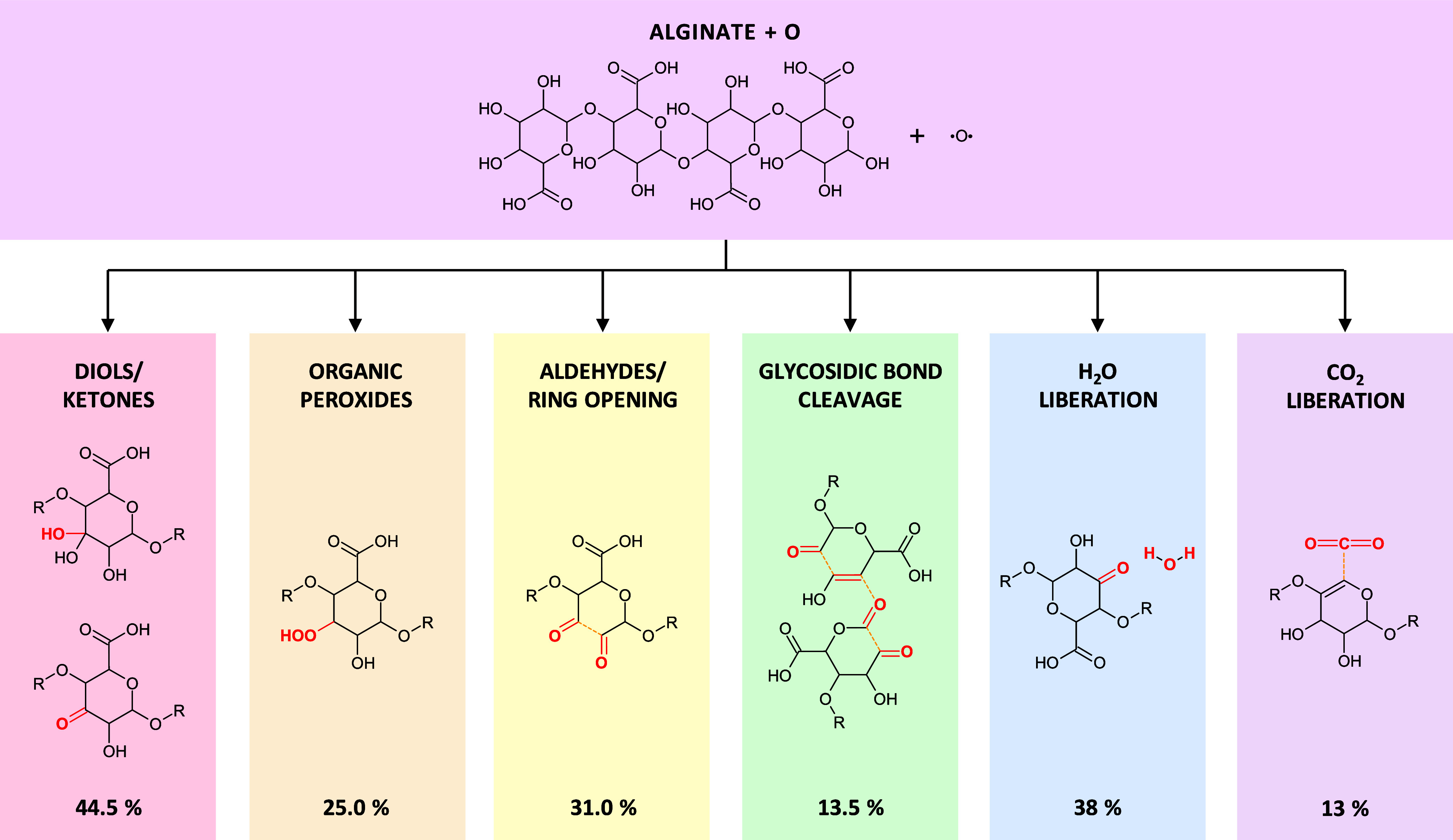
Summary of the main outcomes and associated probabilities obtained from our reactive MD simulations. Changes are highlighted by red atoms/bonds. Broken bonds are represented as dashed orange lines. The probabilities refer to the occurrence of each event individually among the 200 simulated trajectories. Since multiple events can occur simultaneously within a single trajectory, the reported percentages are not mutually exclusive and therefore sum to more than 100%.

Our simulations reveal that the majority of the potential reactions between O atoms and the alginate tetramer initiate with the abstraction of one or two hydrogen atoms (observed in 93% of the cases), occurring at various carbon (in 39.5% of the cases), oxygen (in 35.5% of the cases), or both carbon and oxygen atoms (in 18.0% of the cases) in the molecule. Figure 3 illustrates the reactions involving H-abstraction from one carbon (Figure 3a), one oxygen (Figure 3b), one carbon and one oxygen (Figure 3c) and two oxygen atoms (Figure 3d) of the tetramer molecule. These reactions can lead to different outcomes.

**Figure 3:**
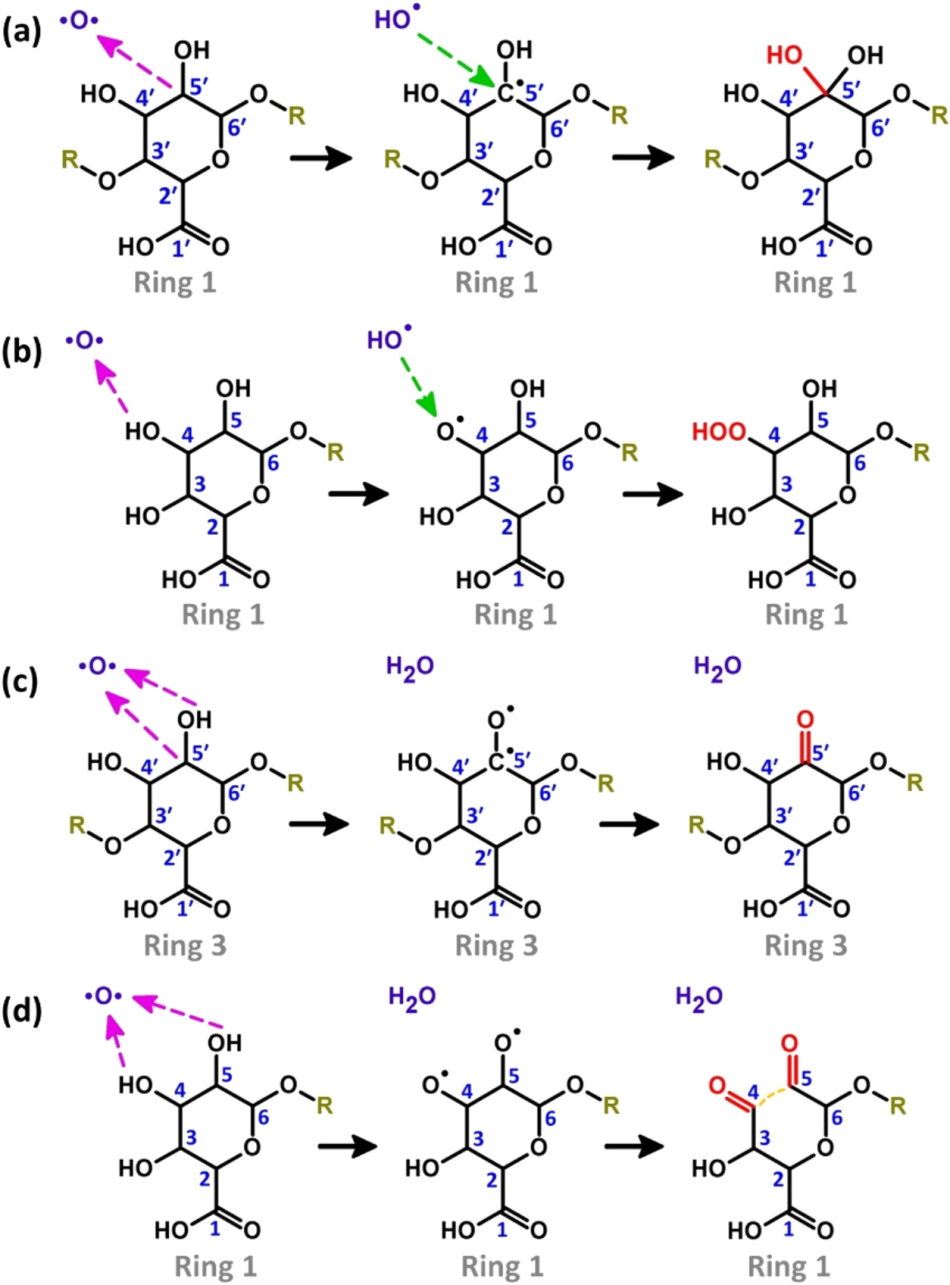
Reaction mechanisms depicting H-abstraction from C or O atoms in various regions of the alginate tetramer molecule (showing only one monomer unit, for simplicity), resulting in the formation of hydroxyl (a), hydroperoxide (b), and carbonyl groups (c, d). Magenta dashed arrows represent H-abstraction reactions, while green dashed arrows indicate OH addition reactions. Newly formed functional groups are highlighted in red, and the breaking of the C–C bond in (d), corresponding to the opening of Ring 1 (cf. Figure 1), is denoted by an orange dashed line. Note that similar reaction mechanisms shown in (a–d) are observed in other monomer units of the alginate tetramer (see Table S1 of the SI).

The main outcome, in terms of occurrence, is the formation of hydroxyl groups (α-hydroxycarboxylic acids or geminal diols), and ketone groups. Hydroxyl groups are formed via H-abstraction from a C atom in a 6-membered ring, generating a C-centered radical and an OH radical, followed by the recombination of these two. The ketone groups are formed via H-abstraction from neighboring C and O atoms, followed by recombination through the formation of a C=O double bond. These products can interconvert between each other via addition/removal of a water molecule and all together account for 44.5% of the total outcomes (see Figure 2). Examples of these reactions are reported in Figure 3a,c and correspond to mechanisms No. 14 and 33, respectively (Table S1, SI). All the products with a keto group within the same ring are also in equilibrium between themselves via keto–enol tautomerism.

The second most frequent outcome is the formation of one or two aldehyde groups with simultaneous opening of one of the 6-membered rings. This can occur via two H-abstractions from two different OH groups, leaving two O-centered radicals. A single C–C bond is then homolytically broken, forming two C-radicals that recombine with the two O-radicals to form two carbonyl groups. An example is reported in Figure 3d and corresponds to mechanism No. 39 (Table S1, SI). Putting all together, the mechanisms leading to this outcome account for 31% of the total (see Figure 2).

Another outcome, that occurred in 25% of the runs, is the formation of peroxides (R_1_–O–O–R_2_, R_1_–O–O–H or R_1_–O–O^–^, see Figure 2). These species are generally formed by H-abstraction from an O atom, generating a OH radical and a O-centered radical, followed by the recombination of these two. In a few cases the recombination is not immediate, leaving a reactive O-radical in the molecule. An example of the formation of peroxides is reported in Figure 3b and corresponds to mechanism No. 22 (Table S1, SI).

Other common events that occur in many mechanisms are the liberation of H_2_O molecules, in 38% of the runs, and CO_2_ molecules in 13% (see Figure 2). H_2_O molecules are formed when a OH radical, generated by H-abstraction by the impacting O atom in any part of the model molecule, takes a second proton from another C or O atom in the molecule. In most cases, the H-abstractions are in positions close to each other in the biopolymer and therefore, the two radicals that are formed, tend to recombine. CO_2_ liberation derives, in most cases, from H-abstraction from a carboxylic group.

Other H-abstraction reaction mechanisms can lead to the formation of other minor products as, for example, carbenes, C=C double bonds and radical sites in the molecule, shortening or widening of its sugar rings, as well as fragmentation of the molecule into other small parts. Note that many of these compounds exhibit instability, suggesting that if they arise during NTP treatment, they are likely to undergo further reactions to form more stable products (see Discussion section below).

One of the important reaction mechanisms that ultimately leads to fragmentation of the alginate tetramer molecule is the cleavage of the glycosidic bond between the sugar monomers. Figure 4 demonstrates an H-abstraction reaction mechanism that induces the formation of carbonyl groups and the breakage of C–O and C–C bonds (see reaction No. 74 in Table S1, SI). Specifically, H-abstraction from C_5′_–OH by an O atom results in the formation of an OH radical, C_5′_=O and C_6′_=O double bonds and the dissociation of C_5′_–C_6′_ and C_9′_–O bonds, the latter being the glycosidic bond. Subsequent H-abstraction by the OH radical leads to the formation of stable C_9′_=C_10′_ and C_11′_=O double bonds, resulting in the breaking of the C_10′_=C_11′_ bond (see Figure 4).

**Figure 4:**
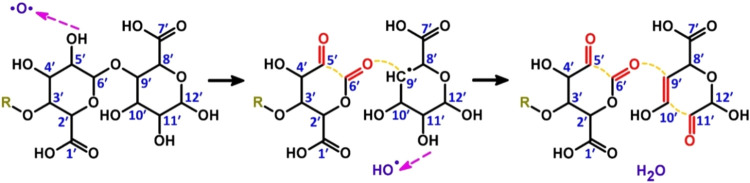
H-abstraction by the O atom, resulting in the cleavage of the C_6′_–O–C_9′_ glycosidic bond and subsequent fragmentation of the alginate tetramer molecule. Magenta dashed arrows indicate the H-abstraction reactions. Newly formed carbonyl groups are depicted in red, while the breaking of the C–C and C–O bonds is illustrated by an orange dashed line.

This subsequently leads to the opening of Rings 3 and 4 (cf. Figure 1) and the cleavage of the glycosidic bond, eventually resulting in fragmentation of the alginate molecule.

## Discussion

A good insight in the mechanisms upon interaction between NTP components and organic (macro)­molecules at a molecular level is necessary to understand and control their effects on biological systems (cells, tissues, etc.) for plasma medicine applications. However, this is not an easy task, since during the plasma treatment of a water solution containing organic matter (small molecules and/or macromolecules), the chemical complexity of the system can be very high.^4,8,11^ In fact, during a plasma treatment, any organic molecule can be oxidized step-by-step, until full mineralization.^36^ All the intermediate products of these oxidation reactions can be produced and, according to their reactivity, can accumulate in solution or further react to generate other molecules. These molecules can also affect the stability of plasma-generated RONS.^3,8^ Given the high complexity and reactivity of these system, a full experimental analysis of the solution, for example by mass spectrometry, preceded by chromatographic separation, is very difficult and sometimes impossible. Computational chemistry plays a fundamental role in this context since it allows to study all possible interaction, at molecular level, between organic matter and reactive species generated by plasma.

In a previous work, we reported the modification caused by NTP treatment on alginate in its solution and hydrogel form.^8^ This was done by rheology, size exclusion chromatography, scanning electron microscopy and mass spectrometry. The experimental results were also supported by a computational study using glucuronate as model molecule (alginate “monomer”), as already mentioned above. The computational results helped explaining the experimental outcomes and were fundamental in understanding the mass spectrometry data. However, the monomer model could not predict glycosidic bond cleavage in alginate upon plasma interaction. Therefore, in this study, we developed a tetrameric model consisting of two guluronic and two mannuronic acid residues linked by β­(1,4) glycosidic bonds (Figure 1). This model allows to study the interaction of plasma-generated short-lived reactive species with all types of chemical bonds existing in an alginate polysaccharide chain.

The results of our new study confirm that, in most cases, the first step of the alginate interaction with plasma-generated short-lived species (O atoms or OH radicals) is the abstraction of H atoms from the biopolymer, either connected to C atoms (CHR_2_ groups from the 6-membered rings) or O atoms (hydroxyl groups or carboxylic groups). This leaves unstable C- or O-radicals that rearrange to form more stable species.

The main outcome, which occurred in 44.5% of the runs (see Figure 2), is the formation of geminal diols or ketones (Figure 3a,3c). These two species are in equilibrium via addition or elimination of a water molecule (Scheme 1), the equilibrium being more shifted toward the keto-form due to steric hindrance. Within the same ring, most of the diol/keto forms are also in equilibrium between themselves via keto–enol tautomerism (Scheme 2).

**Scheme 1:**
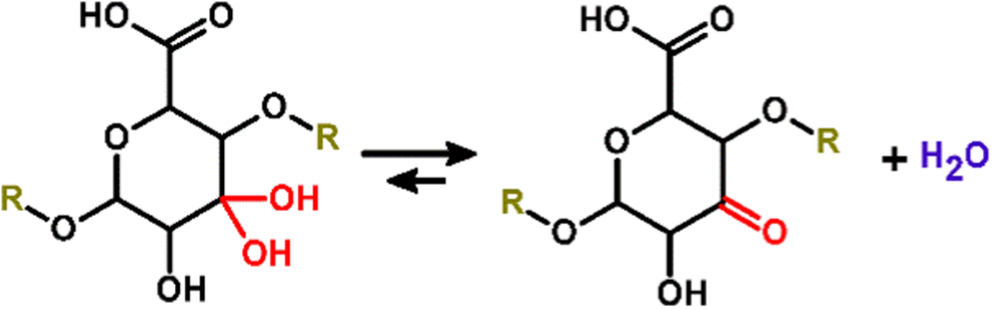
Hydrate-Keto Equilibrium

**Scheme 2:**
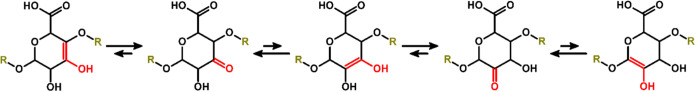
Keto-Enol Tautomerism

The second outcome, in terms of occurrence, in 31% of the runs (see Figure 2), is the formation of two aldehyde groups in neighboring positions on one ring, with subsequent ring opening (Figure 3d). In this case, hydration equilibria and aldehyde-enol tautomerism are also possible.

Another common outcome, with 25% of occurrence (see Figure 2), is the formation of organic peroxides (Figure 3b). These products are expected to be unstable in solution and capable of oxidizing other molecules, such as H_2_O or NO (which are commonly generated during plasma treatment in the presence of air), resulting in their reduction to the original alcohol groups (Scheme 3). This reaction is interesting in a plasma-aqueous system, because it can be the source of secondary long-lived species in the liquid phase, such as H_2_O_2_ and NO_2_.

**Scheme 3:**
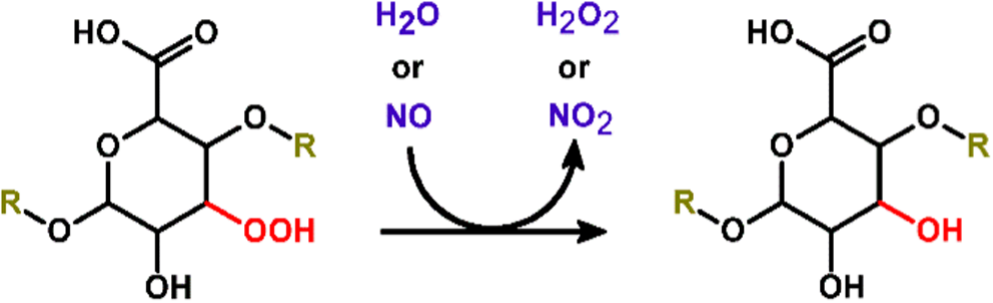
Reduction Reaction of Organic Peroxides

Many reactions occurring upon interaction of O atoms with alginate also involve the liberation of H_2_O or CO_2_ molecules (occurrence of 38 and 13%, respectively, see Figure 2). CO_2_ is formed from the carboxylic groups in the alginate chain. Thus, in 13% of the runs we observed the reduction of the number of carboxylic groups in the main chain. In alginate, carboxyl groups play a crucial role in both the buffering effect of the biopolymer (p*K*_a_ 1.5–3.5) and its cross-linking ability through the formation of ionic bonds with divalent cations in solution. A reduction in the number of carboxylic groups in the polymer chain can therefore weaken the buffering capacity of alginate and reduce its cross-linking potential.

Another key finding of our study is the observation of glycosidic bond cleavage in alginate during plasma treatment (Figure 4). We identified several cases (13.5% of the runs, see Figure 2) where alginate oxidation led to the fragmentation of the polymeric chain. Since all residues in the chain share a common chemical structure, cleavage can potentially occur at any position, resulting in a decrease in molecular weight and a highly polydisperse distribution.

Additionally, our calculations reveal other minor reaction pathways that produce various degradation products, as detailed in Table S1 of the SI. Many of these compounds appear to be unstable, suggesting that if they form during NTP treatment, they are likely to undergo further reactions to yield more stable products.

Comparing the results of this study with our previous calculations,^8^ we can conclude that extending the model from an alginate monomer (glucuronate) to a tetramer yields essentially the same types of outcomes, with only minor variations in occurrence percentages. However, a notable exception is the cleavage of the glycosidic bond, which could not be studied with the simpler monomer model. As mentioned earlier, using a tetramer enables a more comprehensive investigation of the interactions between short-lived reactive species generated by NTP and all possible bonds present in the alginate molecule.

Furthermore, our present findings are consistent with other experimental and simulation studies conducted on similar oligosaccharide systems, as summarized in Table 1. As shown in the table, the main outcomes include glycosidic bond cleavage between sugar monomers, the formation of functional groups (e.g., carbonyl, aldehyde, and hydroxyl groups), bond dissociation, and structural changes such as ring opening and chain shortening.

**Table 1:**
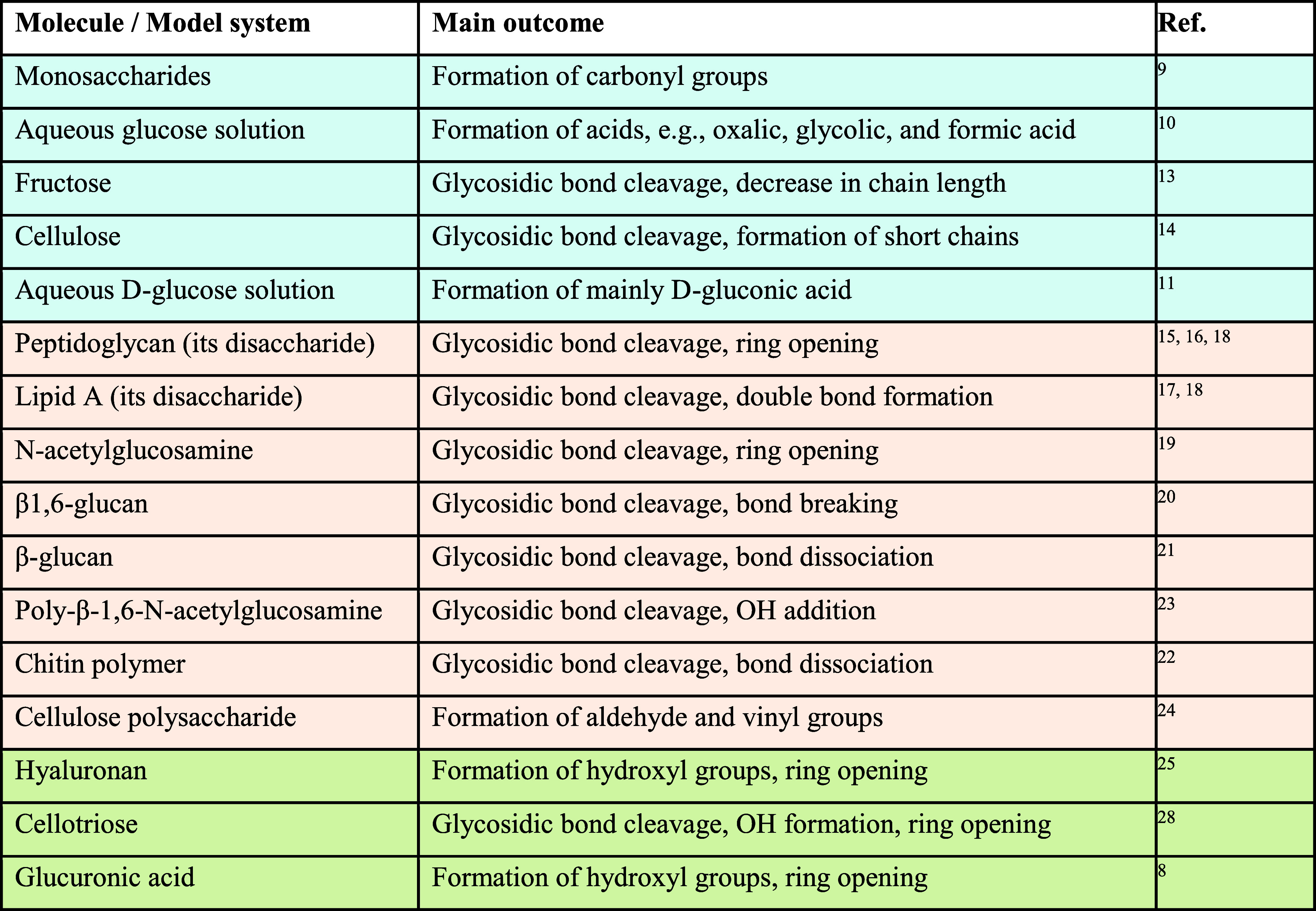
Summary of Experimental (Light Blue), Simulation (Light Orange), and Combined (Light Green) Studies on the Effects of NTP on Various Oligosaccharides

The most effective way to validate results from a computational study is by comparing them with experimental data. All major outcomes presented in this study can be directly or indirectly linked to experimental evidence reported in literature. Figure 5 provides a concise summary of the main correlations between our present computational results and the experimental findings from our previous study.

**Figure 5:**
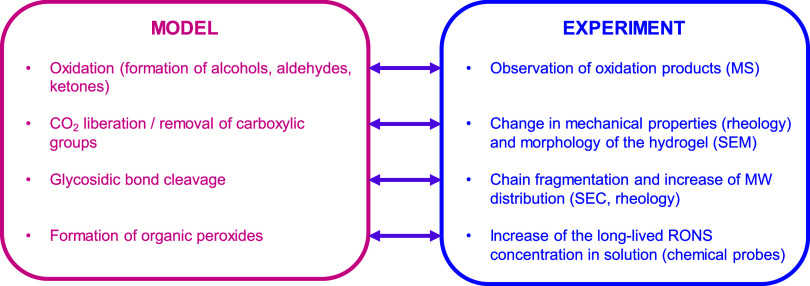
Comparison of outcomes from our computational modeling (left) and experimental observations (right). MS: mass spectrometry; SEM: scanning electron microscopy; MW: molecular weight; SEC: size-exclusion chromatography; RONS: reactive oxygen and nitrogen species.

First, we observed by mass spectrometry the oxidation of alginate chain and the generation of products with keto groups or enols (keto–enol tautomerism reported in Scheme 2). Similar results were also observed by studying the mass spectra of plasma-treated glucose solutions.^11^ Second, changes in mechanical properties (storage modulus) and morphology of alginate hydrogels following plasma treatment, observed by rheology and scanning electron microscopy measurements, respectively, suggested a lower cross-linking degree, in line with a reduction of the carboxylic groups in the alginate chains.^8^ Third, the cleavage of glycosidic bonds supports the evidence that plasma treatment is able to fragment alginate chains, generating fragments with a wide distribution of molecular weight. This was observed by size-exclusion chromatography and by viscosity measurements, since the viscosity of a biopolymer solution can be correlated with its average molecular weight.^37^ The chain fragmentation in solution due to plasma treatment was also reported for other polysaccharides, like methylcellulose,^38^ cellotriose^28^ and starch^39^ and for proteins.^40^ Finally, the formation of organic peroxides, which is one of the main outcomes of our calculations, cannot yet be experimentally validated, because their direct experimental observation was not possible yet, due to their high reactivity. However, by assuming their formation, it is possible to explain the higher production of long-lived plasma-generated reactive species (H_2_O_2_, NO_2_^–^) in solutions containing biopolymers, if compared with the solvent-only counterparts.^6,8,38^ This may occur via the mechanism reported in Scheme 3.

## Conclusions

In this study, we employed reactive molecular dynamics simulations to investigate the atomic-scale interactions of reactive oxygen species, specifically O atoms, with an alginate tetramer. Our results reveal that the primary reaction pathway involves H-abstraction, occurring in 93% of the cases, leading to subsequent oxidation and structural modifications, such as hydroxyl and ketone formation (44.5%), glycosidic bond cleavage (13.5%), and ring opening (31%). Our simulations also identify the formation of organic peroxides (25%), liberation of H_2_O (38%) and CO_2_ (13%), as well as the reduction of carboxyl groups, which are critical for the cross-linking properties of alginate.

A key outcome of our study is the direct observation of glycosidic bond cleavage, which results in fragmentation of the alginate polymer. This finding aligns with prior experimental evidence showing molecular weight reduction and lower viscosity in plasma-treated polysaccharides. Our computational results also highlight the role of oxidation in altering the chemical composition and stability of the alginate structure, with implications for its functional properties in biomedical applications.

By extending our previous computational models from monomeric glucuronate to a tetrameric alginate structure, this study provides a more comprehensive understanding of how plasma-generated reactive species interact with biopolymers. The identified reaction mechanisms contribute to explaining experimental observations, including oxidation-induced changes in hydrogel properties and the presence of degradation products in plasma-treated solutions.

Overall, our findings enhance the mechanistic understanding of plasma-biomaterial interactions at the molecular level. This knowledge is essential for optimizing plasma treatment conditions to tailor the properties of alginate-based materials for biomedical applications, such as wound healing and drug delivery.

## Supplementary Material







## Data Availability

All the data used in this work is uploaded as Supporting Information. No proprietary software was used.

## Notes

Equilibration of the model used for the molecular dynamics calculations; table containing all the results of the molecular dynamics calculations (PDF)
Data input files: Starting geometries and input files used to obtain the results (ZIP)
SMILES: Simplified Molecular Input Line Entry System for all the outputs of the MD calculations (XLSX)
