# Supplementary material for: Modeling Plasma-Induced Modifications in Alginate Biopolymers at the Atomic Scale
Source: J Phys Chem C Nanomater Interfaces. 2025 May 1;129(19):8927–36. doi: 10.1021/acs.jpcc.5c01565 (PMC12133024; doi:10.1021/acs.jpcc.5c01565)
Supplement: Supplementary file 1 [file jp5c01565_si_001.pdf]

SUPPORTING INFORMATION

Modeling Plasma-Induced Modifications in Alginate Biopolymers at the Atomic Scale

Maksudbek Yusupov[a,b,c,1,*], Francesco Tampieri[d,e,f,1,*], Shakhrizoda Matnazarova[g], Nosir Matyakubov[h], Cristina Canal[d,e,f], Annemie Bogaerts[c]

[a] Institute of Fundamental and Applied Research, National Research University TIIAME, Tashkent 100000, Uzbekistan
[b] Department of Information Technologies, Tashkent International University of Education, Tashkent 100207, Uzbekistan
[c] Research group PLASMANT, Department of Chemistry, University of Antwerp, Antwerp 2610, Belgium
[d] Biomaterials, Biomechanics and Tissue Engineering Group, Department of Materials Science and Engineering and Institute for Research and Innovation in Health (IRIS), Universitat Politècnica de Catalunya - BarcelonaTech (UPC), Barcelona 08019, Spain
[e] Barcelona Research Centre in Multiscale Science and Engineering (CCEM), UPC, Barcelona 08019, Spain
[f] Centro de Investigación Biomédica en Red de Bioingeniería, Biomateriales y Nanomedicina (CIBER-BBN), Instituto de Salud Carlos III, Madrid 28029, Spain
[g] Arifov Institute of Ion-Plasma and Laser Technologies, Academy of Sciences of Uzbekistan, Tashkent, Uzbekistan
[h] Department of Physics, Urgench State University, Urgench 220100, Uzbekistan

* Corresponding author.
[1] Shared first author.
e-mails: maksudbek.yusupov@uantwerpen.be (M. Yusupov), francesco.tampieri@upc.edu (F. Tampieri)

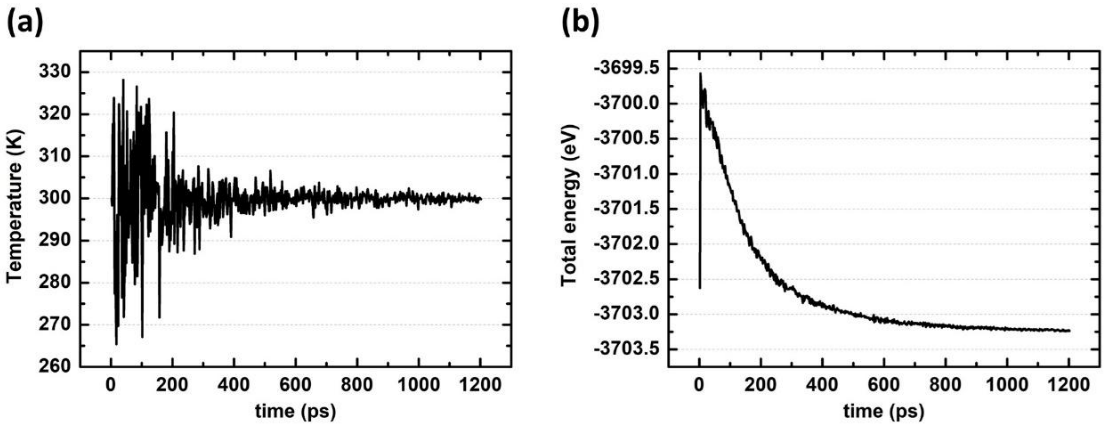

Figure S1. Time evolution of the temperature and total energy of the alginic acid model system, demonstrating that the equilibration time of 1200 ps was sufficient for obtaining a well-thermalized structure.

Table S1. Overview of all reaction mechanisms observed in the DFTB-MD simulations following the interaction of O atoms with the alginic acid tetramer. The numbering of the C atoms corresponds to Figure 1 in the main text. Note that most of the reactions are initiated by H-abstraction from different C or O atoms (1-75) and the last ones (76-85) are initiated by O addition. Color code: green = events that happen with frequency higher than 2.5%; yellow = events that happen with frequency between 2.5 and 1.5% (values included); white = events that happen with frequency lower than 1.5%.

No.	H-abstraction	Number of events	Description	Structure	%
1	C_2H	5	C_2-OH is formed<br>α-hydroxy acid<br><br>$C_{24}H_{34}O_{26}$ (738 Da)	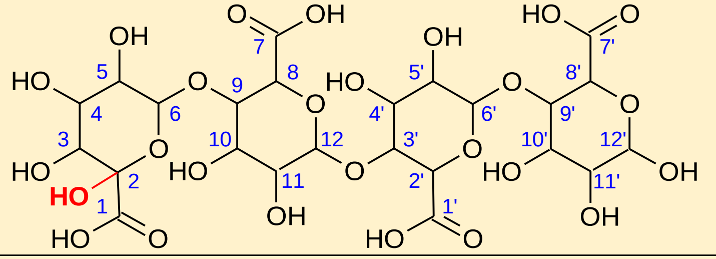	2.5
2	$C_{2'}H$	4	$C_{2'}$-OH is formed<br>α-hydroxy acid<br><br>$C_{24}H_{34}O_{26}$ (738 Da)	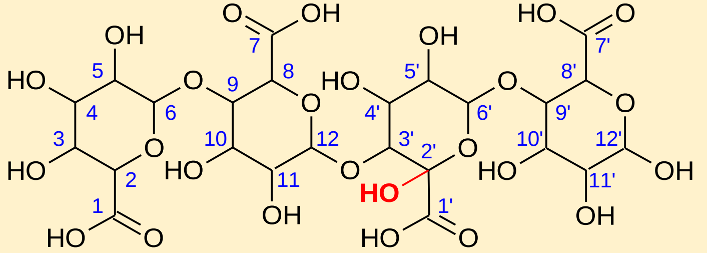	2.0
3	C_8H	1	C_8-OH is formed<br>α-hydroxy acid<br><br>$C_{24}H_{34}O_{26}$ (738 Da)	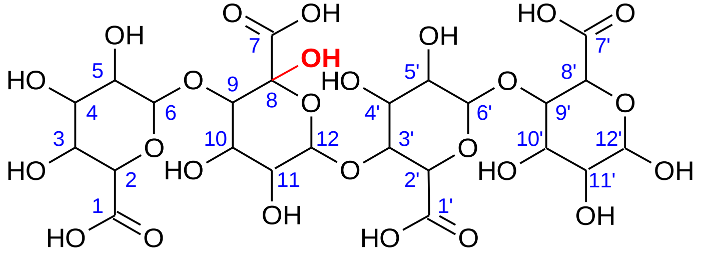	0.5

4	$C_{8'}H$	2	$C_{8'}$-OH is formed<br>α-hydroxy acid<br><br>$C_{24}H_{34}O_{26}$ (738 Da)	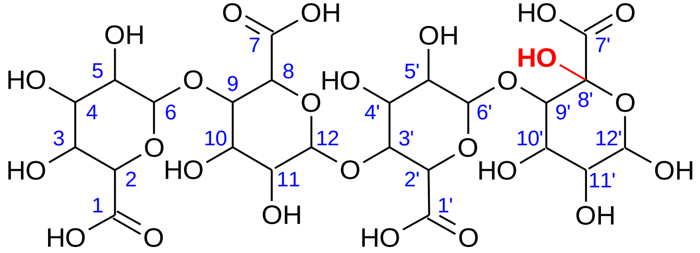	1.0
5	$C_{3'}H$	2	$C_{3'}$-OH is formed<br>geminal diol<br><br>$C_{24}H_{34}O_{26}$ (738 Da)	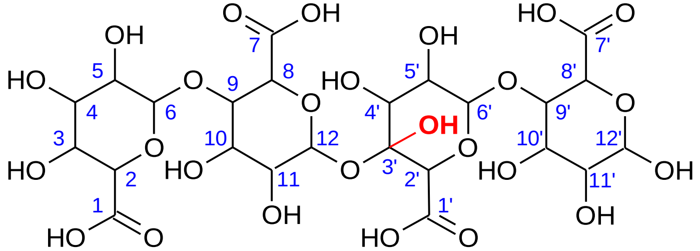	1.0
6	$C_{6}H$	4	C_{6}-OH is formed<br>geminal diol<br><br>$C_{24}H_{34}O_{26}$ (738 Da)	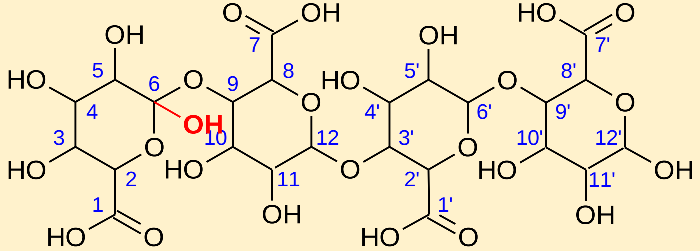	2.0
7	$C_{6'}H$	2	$C_{6'}$-OH is formed<br>geminal diol<br><br>$C_{24}H_{34}O_{26}$ (738 Da)	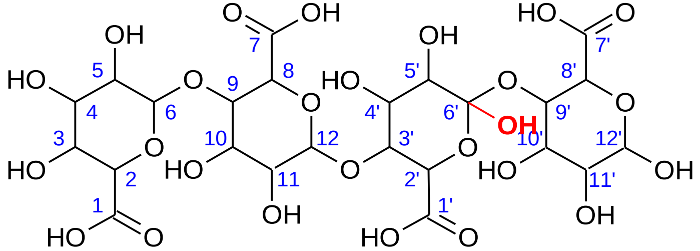	1.0

8	$C_{9'}H$	1	$C_{9'}$-OH is formed geminal diol<br><br>$C_{24}H_{34}O_{26}$ (738 Da)	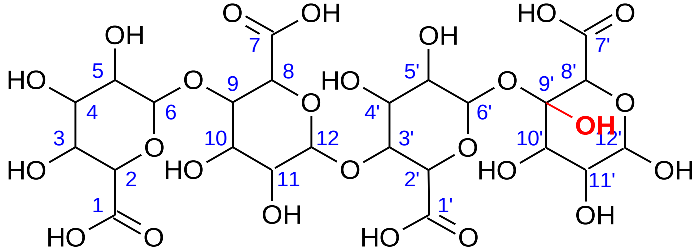	0.5
9	$C_{12}H$	4	C_{12}-OH is formed geminal diol<br><br>$C_{24}H_{34}O_{26}$ (738 Da)	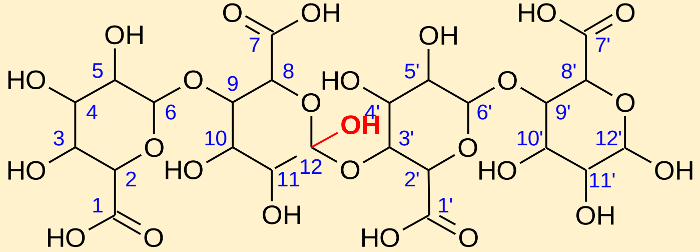	2.0
10	C_3H	6	C_3-OH is formed geminal diol<br><br>$C_{24}H_{34}O_{26}$ (738 Da)	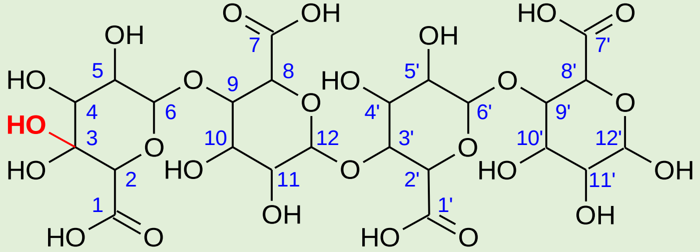	3.0
11	C_4H	6	C_4-OH is formed geminal diol<br><br>$C_{24}H_{34}O_{26}$ (738 Da)	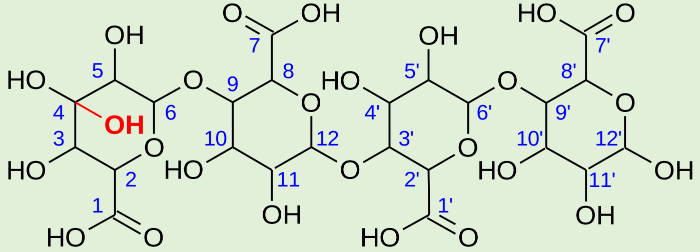	3.0

12	C_5H	3	C_5-OH is formed geminal diol<br><br>$C_{24}H_{34}O_{26}$ (738 Da)	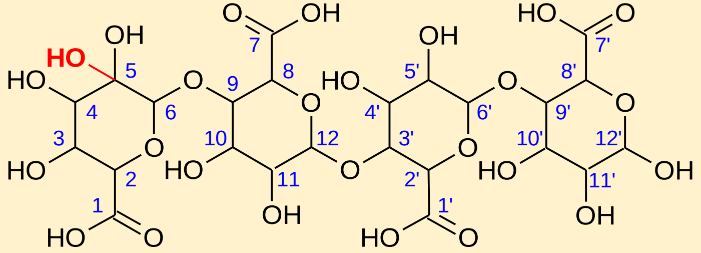	1.5
13	$C_{4'}H$	5	$C_{4'}$-OH is formed geminal diol<br><br>$C_{24}H_{34}O_{26}$ (738 Da)	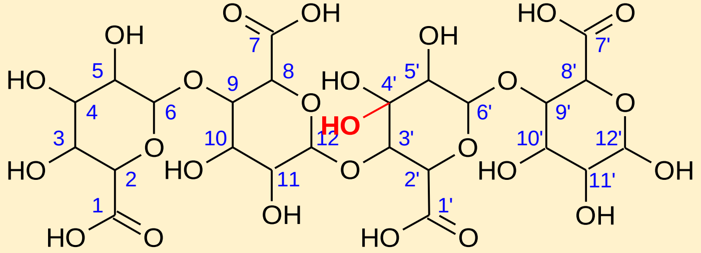	2.5
14	$C_{5'}H$	8	$C_{5'}$-OH is formed geminal diol<br><br>$C_{24}H_{34}O_{26}$ (738 Da)	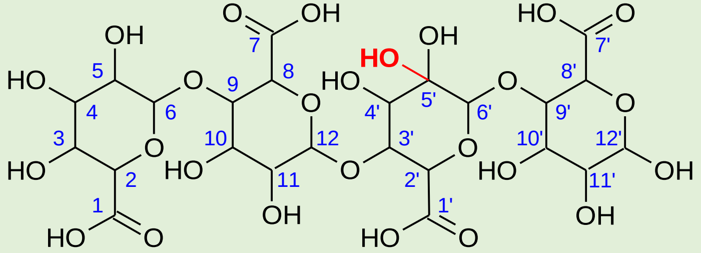	4.0
15	$C_{10}H$	4	C_{10}-OH is formed geminal diol<br><br>$C_{24}H_{34}O_{26}$ (738 Da)	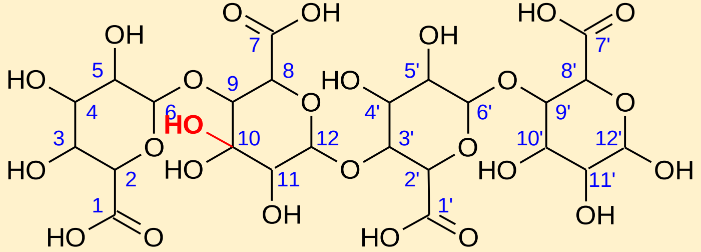	2.0

16	$C_{10'}H$	7	$C_{10'}$-OH is formed geminal diol<br><br>$C_{24}H_{34}O_{26}$ (738 Da)	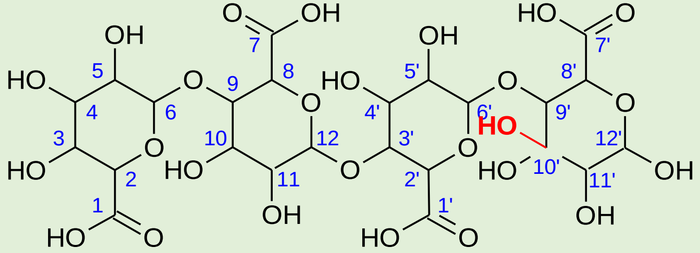	3.5
17	$C_{11}H$	2	C_{11}-OH is formed geminal diol<br><br>$C_{24}H_{34}O_{26}$ (738 Da)	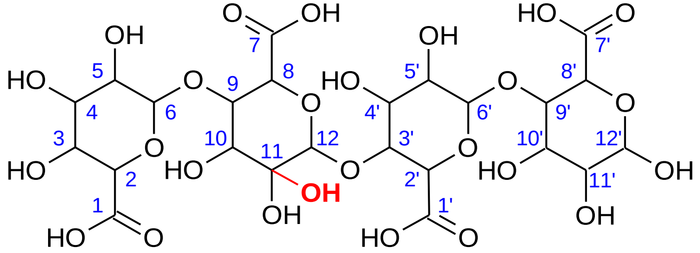	1.0
18	$C_{11'}H$	4	$C_{11'}$-OH is formed geminal diol<br><br>$C_{24}H_{34}O_{26}$ (738 Da)	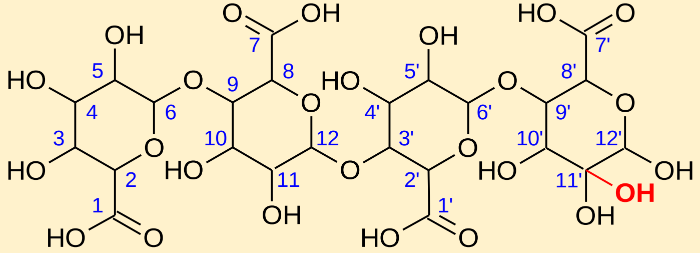	2.0
19	$C_{12'}H$	7	$C_{12'}$-OH is formed geminal diol<br><br>$C_{24}H_{34}O_{26}$ (738 Da)	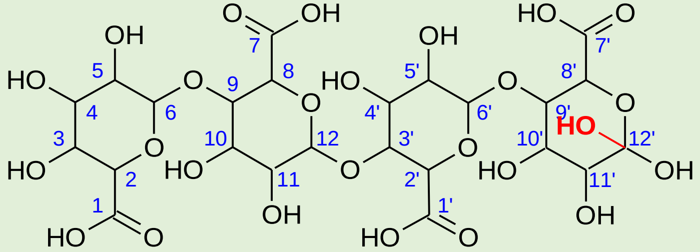	3.5

20	C_1OH	1	C_1O-OH is formed peroxyacid<br><br>$C_{24}H_{34}O_{26}$ (738 Da)	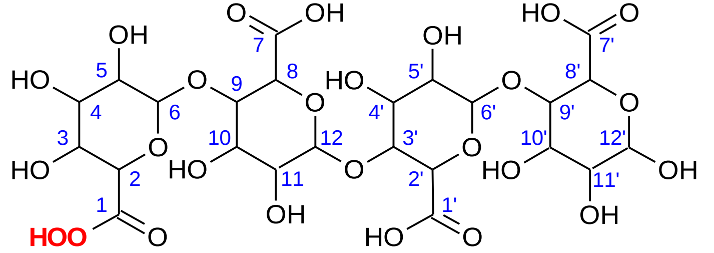	0.5
21	C_3OH	8	C_3O-OH is formed hydroperoxide<br><br>$C_{24}H_{34}O_{26}$ (738 Da)	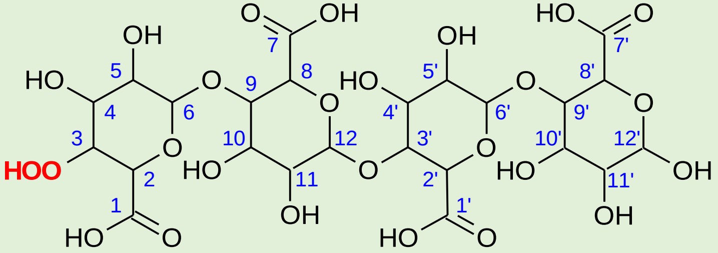	4.0
22	C_4OH	9	C_4O-OH is formed hydroperoxide<br><br>$C_{24}H_{34}O_{26}$ (738 Da)	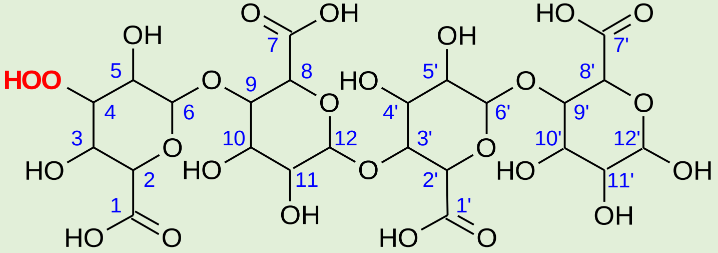	4.5
23	C_5OH	2	C_5O-OH is formed hydroperoxide<br><br>$C_{24}H_{34}O_{26}$ (738 Da)	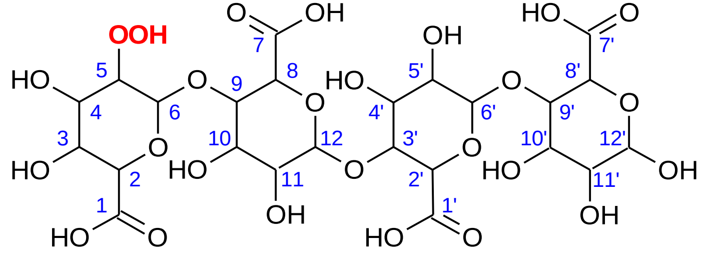	1.0

24	C_7OH	1	C_7O-OH is formed peroxyacid<br><br>$C_{24}H_{34}O_{26}$ (738 Da)	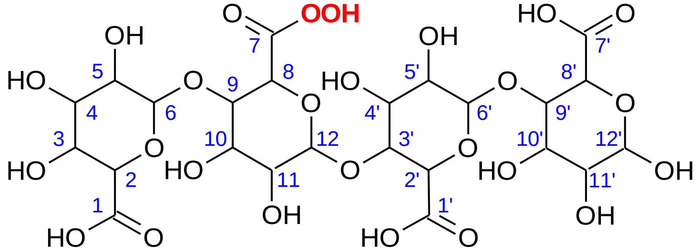	0.5
25	C_{10}OH	1	C_{10}O-OH is formed hydroperoxide<br><br>$C_{24}H_{34}O_{26}$ (738 Da)	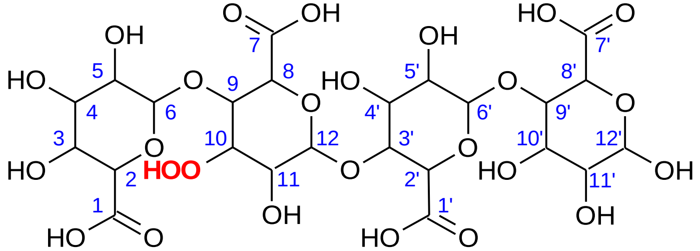	0.5
26	$C_{10'}$OH	2	$C_{10'}$O-OH is formed hydroperoxide<br><br>$C_{24}H_{34}O_{26}$ (738 Da)	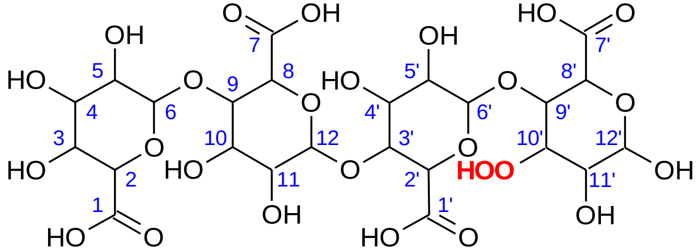	1.0
27	$C_{11'}$OH	2	$C_{11'}$O-OH is formed hydroperoxide<br><br>$C_{24}H_{34}O_{26}$ (738 Da)	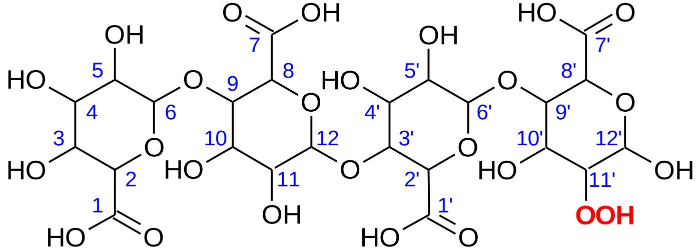	1.0

28	$C_{12'}OH$	1	$C_{12'}O$-OH is formed hydroperoxide<br><br>$C_{24}H_{34}O_{26}$ (738 Da)	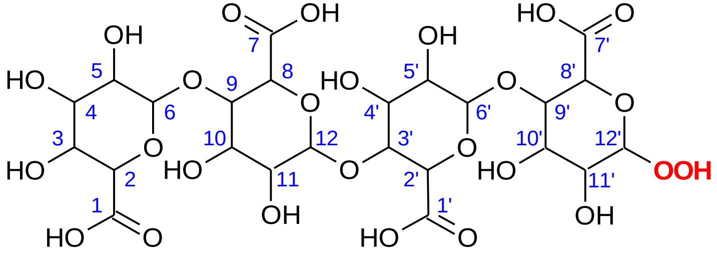	0.5
29	C_3H and C_3OH	1	H_2O and C_3=O is formed ketone group<br><br>$C_{24}H_{32}O_{25}$ (720 Da) + H_2O (18 Da)	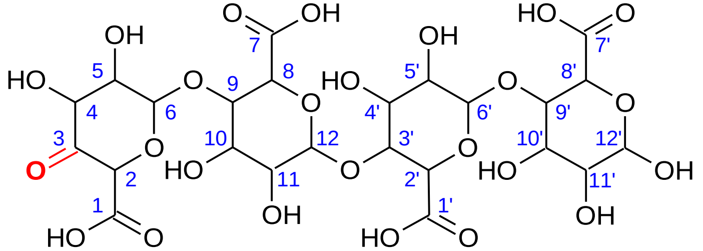	0.5
30	C_4H and C_4OH	1	H_2O and C_4=O is formed ketone group<br><br>$C_{24}H_{32}O_{25}$ (720 Da) + H_2O (18 Da)	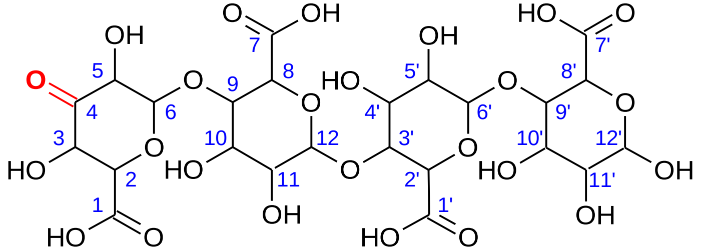	0.5
31	$C_{4'}H$ and $C_{4'}OH$	1	H_2O and $C_{4'}$=O is formed ketone group<br><br>$C_{24}H_{32}O_{25}$ (720 Da) + H_2O (18 Da)	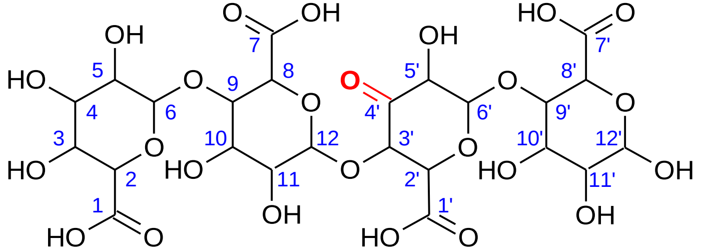	0.5

32	C_5H and C_5OH	1	H_2O and C_5=O is ketone group<br><br>$C_{24}H_{32}O_{25}$ (720 Da) + H_2O (18 Da)	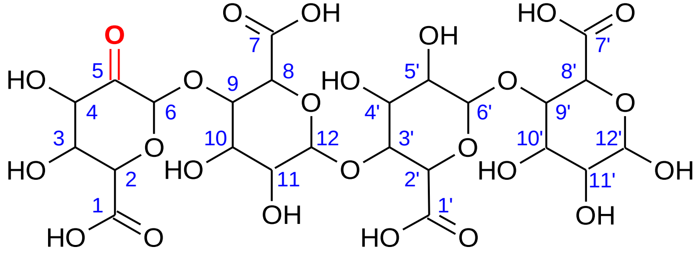	0.5
33	$C_{5'}H$ and $C_{5'}OH$	3	H_2O and $C_{5'}$=O is formed ketone group<br><br>$C_{24}H_{32}O_{25}$ (720 Da) + H_2O (18 Da)	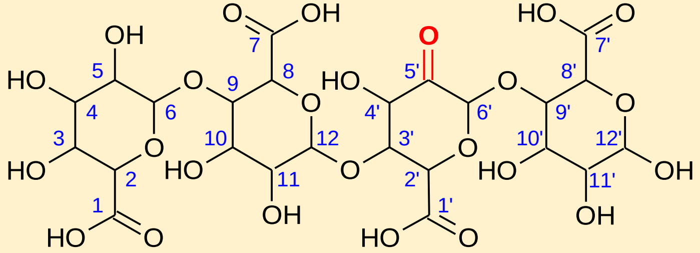	1.5
34	$C_{10}H$ and $C_{10}OH$	1	H_2O and C_{10}=O is formed ketone group<br><br>$C_{24}H_{32}O_{25}$ (720 Da) + H_2O (18 Da)	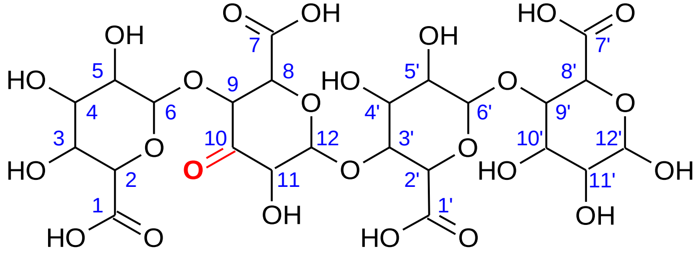	0.5
35	$C_{10'}H$ and $C_{10'}OH$	1	H_2O and $C_{10'}$=O is formed ketone group<br><br>$C_{24}H_{32}O_{25}$ (720 Da) + H_2O (18 Da)	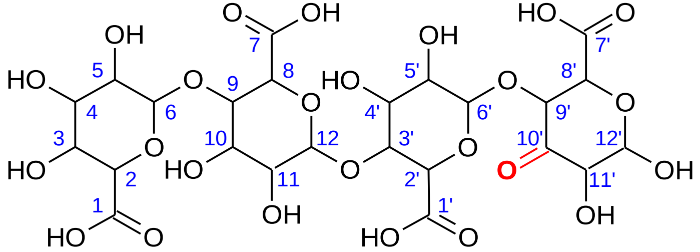	0.5

36	$C_{11}H$ and $C_{11}OH$	1	H_2O and C_{11}=O is formed ketone group<br><br>$C_{24}H_{32}O_{25}$ (720 Da) + H_2O (18 Da)	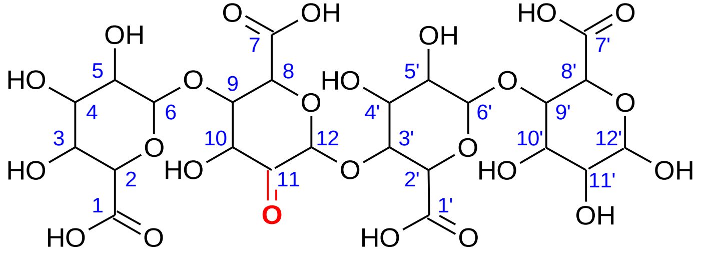	0.5
37	$C_{12'}H$ and $C_{12'}OH$	2	H_2O and $C_{12'}$=O is formed ketone group<br><br>$C_{24}H_{32}O_{25}$ (720 Da) + H_2O (18 Da)	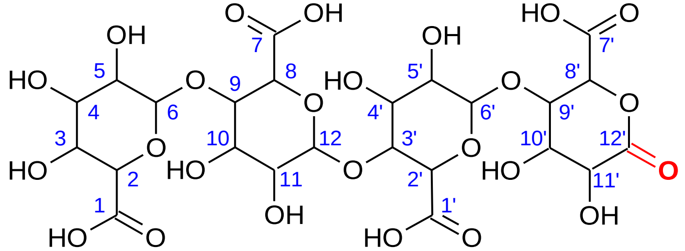	1.0
38	C_3OH and C_4OH	2	C_3-C_4 bond is broken, H_2O, C_3=O and C_4=O are formed<br>2 aldehyde groups, ring 1 opening<br><br>$C_{24}H_{32}O_{25}$ (720 Da) + H_2O (18 Da)	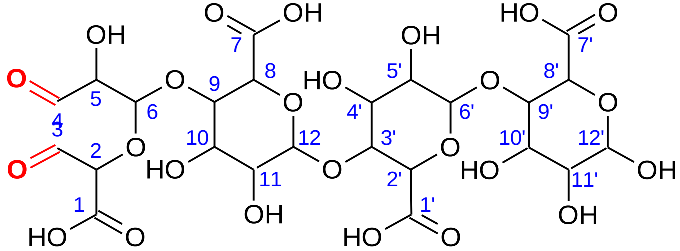	1.0
39	C_4OH and C_5OH	8	C_4-C_5 bond is broken, H_2O, C_4=O and C_5=O are formed<br>2 aldehyde groups, ring 1 opening<br><br>$C_{24}H_{32}O_{25}$ (720 Da) + H_2O (18 Da)	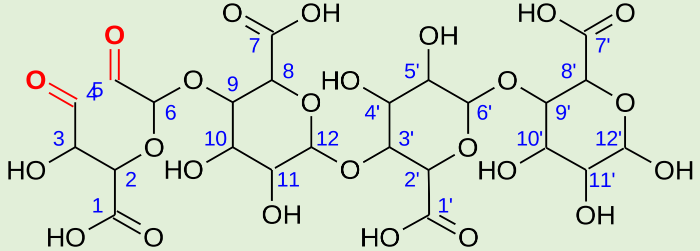	4.0

40	$C_{4'}H$ and $C_{5'}OH$	3	$C_{4'}$-$C_{5'}$ bond is broken, H_2O, $C_{4'}$=O and $C_{5'}$=O are formed<br>2 aldehyde groups, ring 3 opening<br><br>$C_{24}H_{32}O_{25}$ (720 Da) + H_2O (18 Da)	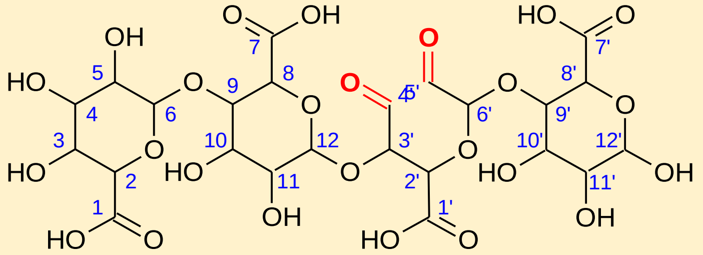	1.5
41	$C_{10}OH$ and $C_{11}OH$	3	C_{10}-C_{11} bond is broken, H_2O, C_{10}=O and C_{11}=O are formed<br>2 aldehyde groups, ring 2 opening<br><br>$C_{24}H_{32}O_{25}$ (720 Da) + H_2O (18 Da)	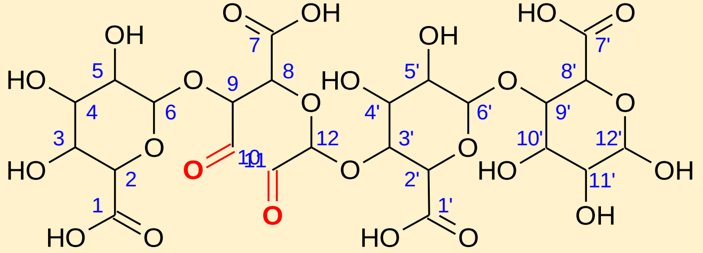	1.5
42	$C_{10'}H$ and $C_{11'}OH$	3	$C_{10'}$-$C_{11'}$ bond is broken, H_2O, $C_{10'}$=O and $C_{11'}$=O are formed<br>2 aldehyde groups, ring 4 opening<br><br>$C_{24}H_{32}O_{25}$ (720 Da) + H_2O (18 Da)	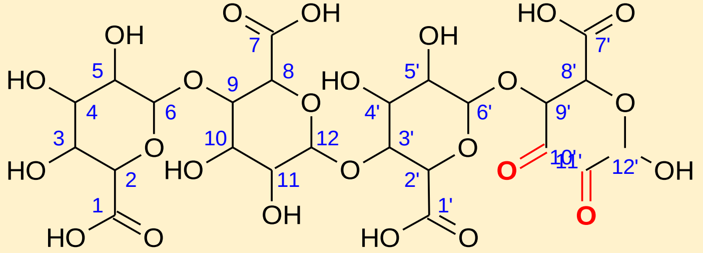	1.5
43	$C_{11'}H$ and $C_{12'}OH$	5	$C_{11'}$-$C_{12'}$ bond is broken, H_2O, $C_{11'}$=O and $C_{12'}$=O are formed<br>2 aldehyde groups, ring 4 opening<br><br>$C_{24}H_{32}O_{25}$ (720 Da) + H_2O (18 Da)	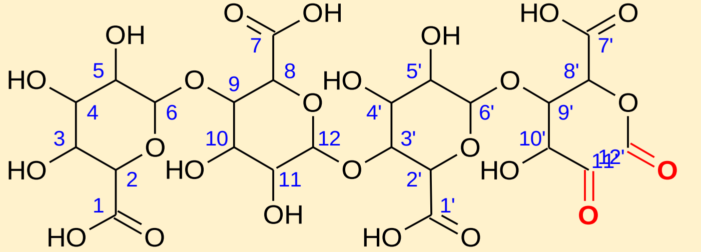	2.5

44	C_2H and C_4OH	1	H_2O is formed, C_3-C_4 bond is broken, C_2=C_3 is formed aldehyde group, ring 1 opening<br><br>$C_{24}H_{32}O_{25}$ (720 Da) + H_2O (18 Da)	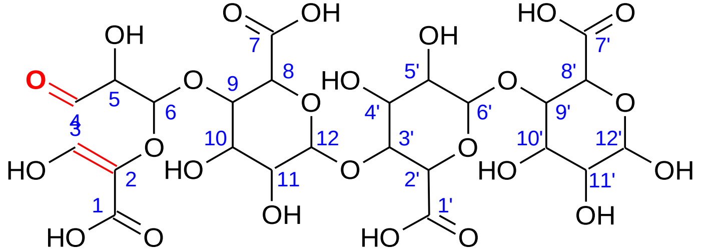	0.5
45	C_3H and C_4OH	1	H_2O is formed, C_3-C_4 bond is broken aldehyde and carbene C_2-(**:**C_3)-O formation, ring 1 opening<br><br>$C_{24}H_{32}O_{25}$ (720 Da) + H_2O (18 Da)	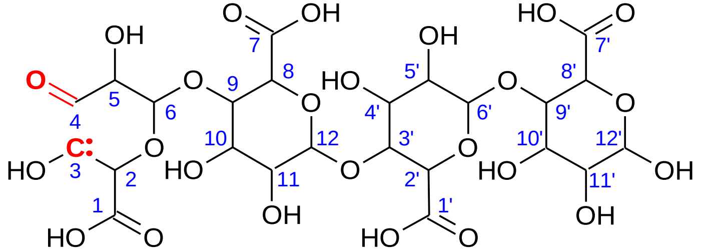	0.5
46	C_4OH and C_5H	1	H_2O is formed, C_4-C_5 bond is broken aldehyde and carbene C_6-(**:**C_5)-O formation, ring 1 opening<br><br>$C_{24}H_{32}O_{25}$ (720 Da) + H_2O (18 Da)	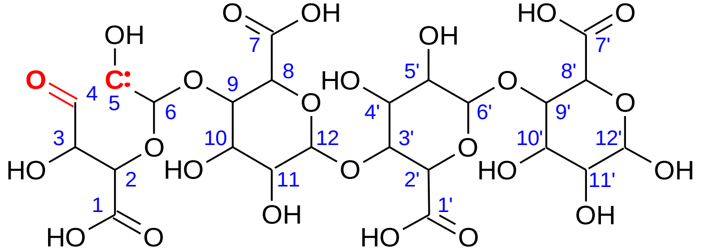	0.5
47	C_2H and C_5OH	1	H_2O is formed, C_5-C_6 bond is broken, C_5=O, $^{\bullet}C_2$ and $^{\bullet}C_6$ are formed aldehyde group, ring 1 opening<br><br>$C_{24}H_{32}O_{25}$ (720 Da) + H_2O (18 Da)	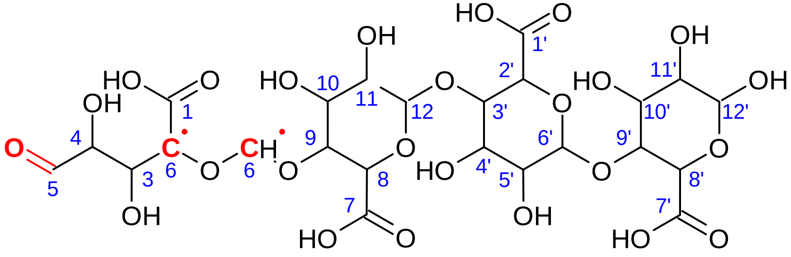	0.5

48	$C_{12'}OH$	3	$C_{8'}O$-$C_{12'}$ bond is broken, $C_{8'}O$-OH is formed<br>aldehyde and hydroperoxide groups, ring 4 opening<br><br>$C_{24}H_{34}O_{26}$ (738 Da)	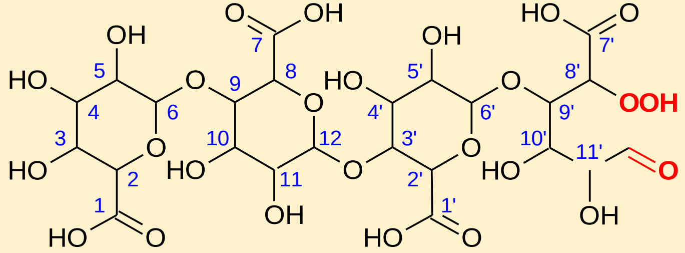	1.5
49	C_5OH and later on C_4OH	1	C_5-C_6 bond is broken, ring 1 opening, H_2O is formed, C_4O-C_6 is formed aldehyde and ether group<br><br>$C_{24}H_{32}O_{25}$ (720 Da) + H_2O (18 Da)	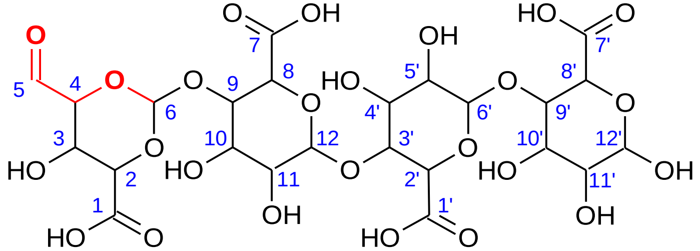	0.5
50	C_5OH and later on C_4OH	1	C_5-C_6 bond is broken (aldehyde formation), ring 1 opening,<br>C_5O-C_6 and C_4O-C_5 are formed<br><br>$C_{24}H_{32}O_{25}$ (720 Da) + H_2O (18 Da)	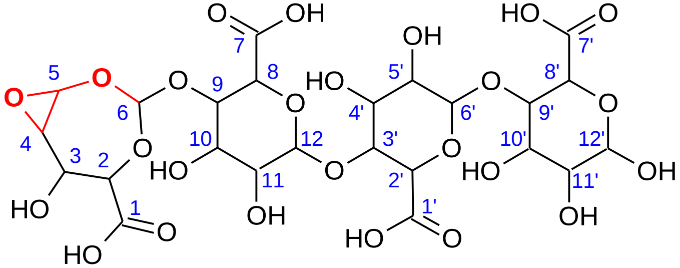	0.5
51	C_1OH and C_2H	2	H_2O formation and CO_2 liberation, carbene C_3=C_2 formation<br><br>$C_{23}H_{32}O_{23}$ (676 Da) + H_2O (18 Da) + CO_2 (44 Da)	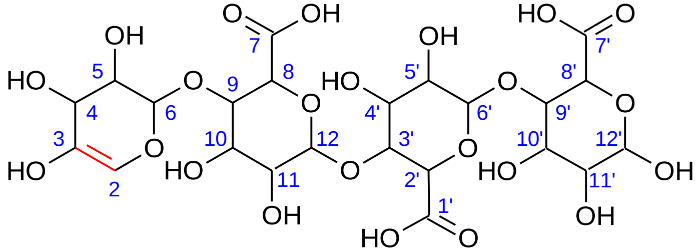	1.0

52	C_1OH and C_6H	1	CO_2 liberation, H_2O formation, $^{\bullet}C_2$ and $^{\bullet}C_6$ formation<br><br>$C_{23}H_{32}O_{23}$ (676 Da) + H_2O (18 Da) + CO_2 (44 Da)	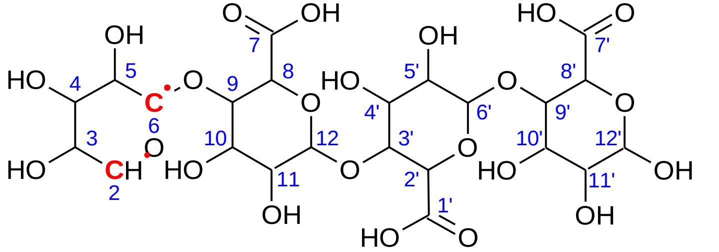	0.5
53	C_1OH and later on $C_{10}OH$	2	H-abs. from C_1OH: CO_2 liberation, C_2O-C_6 bond breaking, ring 1 opening (aldehyde) H-abs. from $C_{10}OH$: H_2O formation, C_9-C_{10} bond breaking, ring 2 opening (aldehyde form.), C_5=C_6 and C_8=C_9 formation, C_4-C_5 and C_8-OC_{12} bonds breaking, H-transfer from C_4OH to $C_{12}O^{\bullet}$ (aldehyde form.)<br><br>$C_{15}H_{22}O_{16}$ (458 Da) + $C_5H_6O_4$ (130 Da) + $C_3H_4O_3$ (88 Da) + H_2O (18 Da) + CO_2 (44 Da)	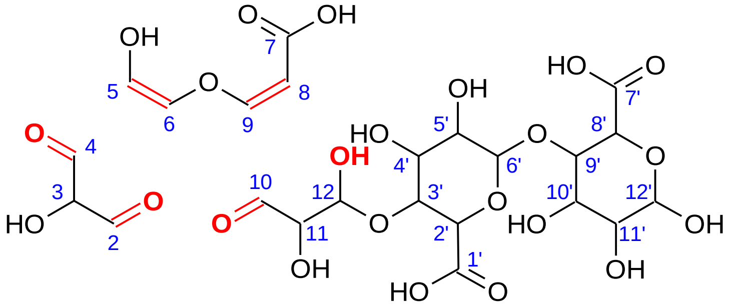	1.0
54	C_1OH and $C_{10}OH$	1	H_2O formation, C_{10}-C_{11} bond breaking (ring 2 opening and aldehyde formation), C_1O-C_{11} bond formation (larger ring)<br><br>$C_{24}H_{32}O_{25}$ (720 Da) + H_2O (18 Da)	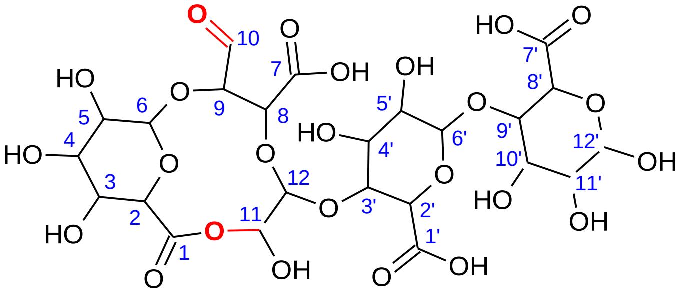	0.5
55	C_1OH and C_3OH	1	H_2O formation, CO_2 liberation, C_3-C_4 bond breaking and C_2-C_4 bond formation (aldehyde and small ring formation)<br><br>$C_{23}H_{32}O_{23}$ (676 Da) + H_2O (18 Da) + CO_2 (44 Da)	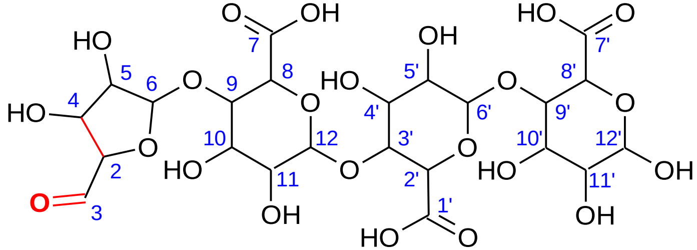	0.5

56	C_3OH and later on C_6H	1	H-abs. from C_3OH: C_3-C_4 bond breaking (aldehyde formation), C_4=C_5 double bond formation, C_5-C_6 bond breaking, H-abs. from C_6H: H_2O formation, 1,2-ethenediol formation, O-(**:**C_6)-O formation<br><br>$C_{22}H_{28}O_{23}$ (660 Da) + $C_2H_4O_2$ (60 Da) + H_2O (18 Da)	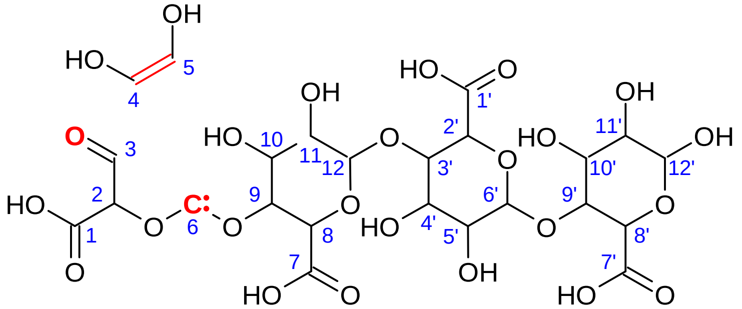	0.5
57	$C_{3'}H$ and $C_{12}H$	1	H_2O formation, 2 C radicals formation<br><br>$C_{24}H_{32}O_{25}$ (720 Da) + H_2O (18 Da)	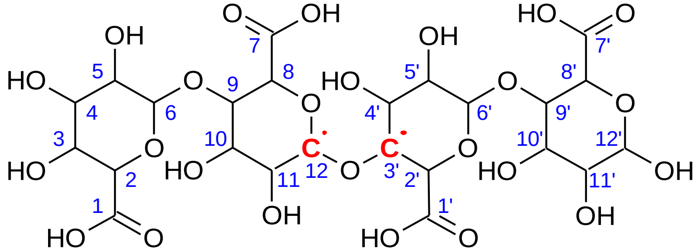	0.5
58	C_7OH and later on $C_{4'}OH$	5	H-abs. from $C_{4'}OH$: $C_{3'}$-$C_{4'}$ bond breaking (aldehyde formation and ring 3 opening) H-abs. from C_7OH: CO_2 liberation, H_2O formation, C_8O-C_{12} bond breaking (ring 2 opening and aldehyde formation),<br><br>$C_{23}H_{32}O_{23}$ (676 Da) + H_2O (18 Da) + CO_2 (44 Da)	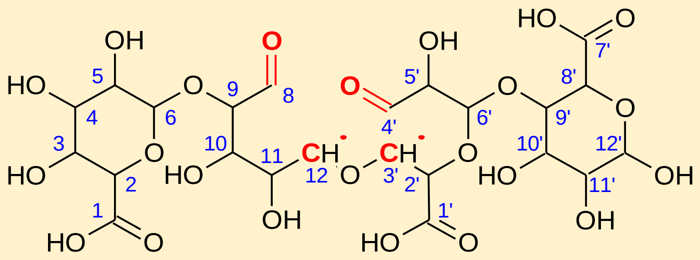	2.5
59	C_5OH and later on C_4OH	1	C_7OH bond formation, H-abstraction from C_4OH by C_7O, C_4-C_5 bond breaking, (ring 1 opening, aldehyde and triol formation)<br><br>$C_{24}H_{34}O_{26}$ (738 Da)	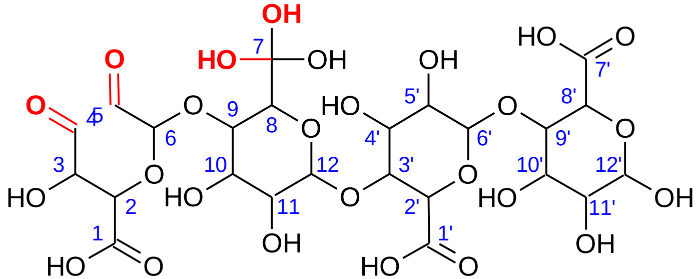	0.5

60	C_5OH and $C_{10}H$	1	H_2O formation, C_5-O-C_{10} formation (extra ring formation)<br><br>$C_{24}H_{32}O_{25}$ (720 Da) + H_2O (18 Da)	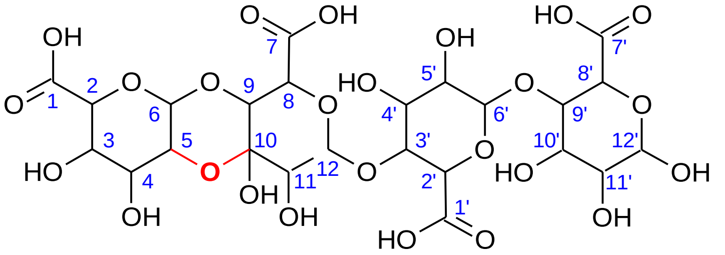	0.5
61	$C_{5'}OH$ and $C_{10'}OH$	1	H_2O formation, $C_{5'}$-$C_{6'}$ bond breaking (ring 3 opening, aldehyde formation), $C_{6'}$-O-$C_{10'}$ formation (small ring formation)<br><br>$C_{24}H_{32}O_{25}$ (720 Da) + H_2O (18 Da)	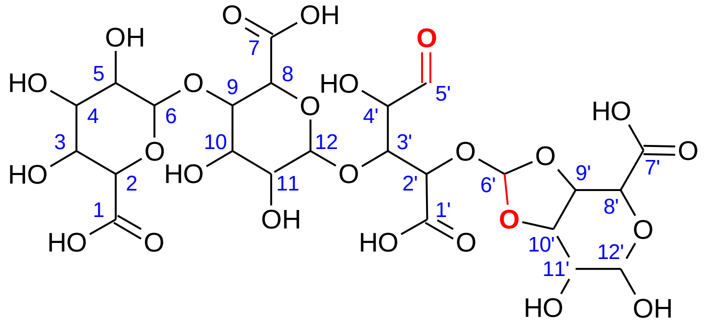	0.5
62	$C_{7'}OH$ and $C_{11'}OH$	2	H_2O formation, CO_2 liberation, $C_{11'}$-$C_{12'}$ bond breaking (ring 4 opening and aldehyde formation), $^{\bullet}C_{8'}$ and $^{\bullet}C_{12'}$ formation<br><br>$C_{23}H_{32}O_{23}$ (676 Da) + H_2O (18 Da) + CO_2 (44 Da)	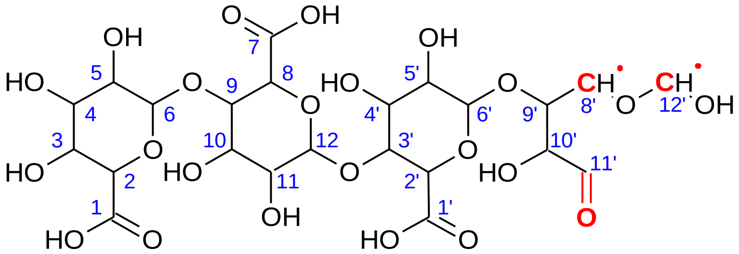	1.0
63	C_7OH and later on $C_{12}H$	2	CO_2 liberation, C_8O-C_{12} bond breaking (ring 2 opening and aldehyde formation), H_2O formation, :C_{12} formation<br><br>$C_{23}H_{32}O_{23}$ (676 Da) + H_2O (18 Da) + CO_2 (44 Da)	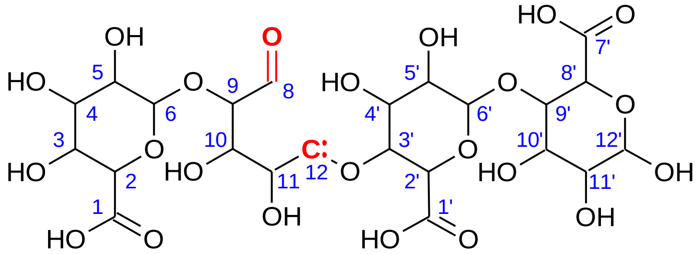	1.0

64	$C_{7'}OH$ and $C_{12'}OH$	1	H_2O formation, CO_2 liberation, $C_{8'}O$-$C_{12'}$ bond breaking (ring 4 opening and aldehyde formation)<br><br>$C_{23}H_{32}O_{23}$ (676 Da) + H_2O (18 Da) + CO_2 (44 Da)	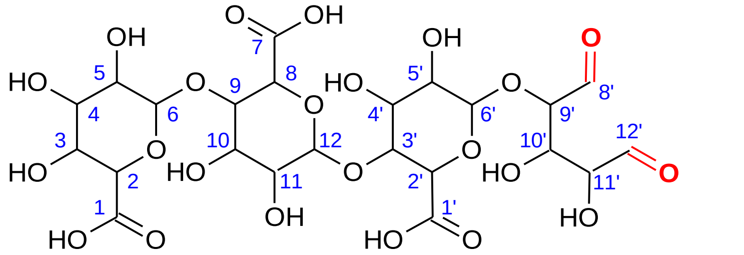	0.5
65	$C_{12'}OH$ and later on $C_{7'}OH$	1	$C_{11'}$-$C_{12'}$ bond breaking (ring 4 opening and aldehyde formation), H_2O formation, CO_2 liberation, $C_{8'}$=$C_{9'}$ double bond formation and $C_{9'}$=$C_{10'}$ breaking (1,2-ethenediol formation)<br><br>$C_{21}H_{28}O_{21}$ (616 Da) + $C_2H_4O_2$ (60 Da) + H_2O (18 Da) + CO_2 (44 Da)	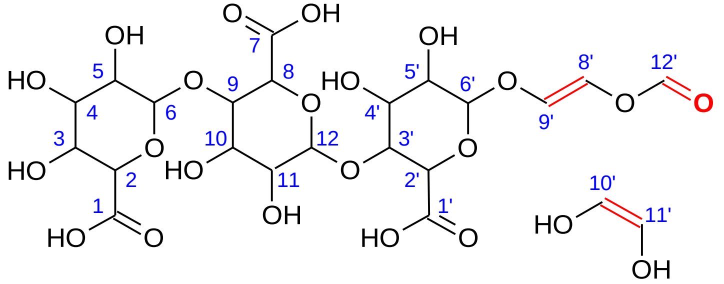	0.5
66	C_4OH and later on $C_{12'}OH$	1	C_3-C_4 bond breaking (ring 1 opening and aldehyde formation), H_2O formation, $C_{8'}O$-$C_{12'}$ bond breaking (ring 4 opening and aldehyde formation), H-transfer from $C_{7'}OH$ to $C_{8'}O$ (CO_2 liberation), $C_{8'}$-$C_{12'}$ bond formation, $^{\bullet}C_3$ formation<br><br>$C_{23}H_{32}O_{23}$ (676 Da) + H_2O (18 Da) + CO_2 (44 Da)	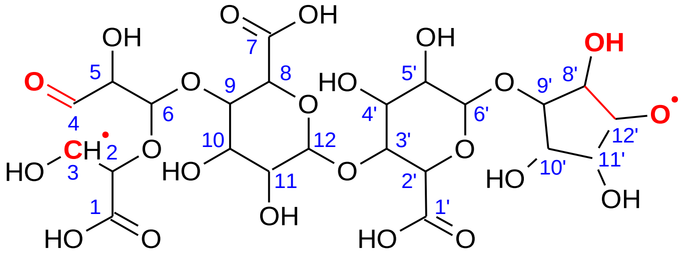	0.5
67	C_7OH and later on C_1OH	2	Binding to C_6-O(=**O**)-C_9, H-abstraction from C_7OH (hydroperoxyl group formation), C_6-OC_9 glycosidic bond cleavage, H-transfer from C_1OH to $C_7O^{\bullet}$ (CO_2 liberation), $^{\bullet}C_2$ and $^{\bullet}C_6$ formation<br><br>$C_{18}H_{26}O_{20}$ (562 Da) + $C_5H_8O_4$ (132 Da) + CO_2 (44 Da)	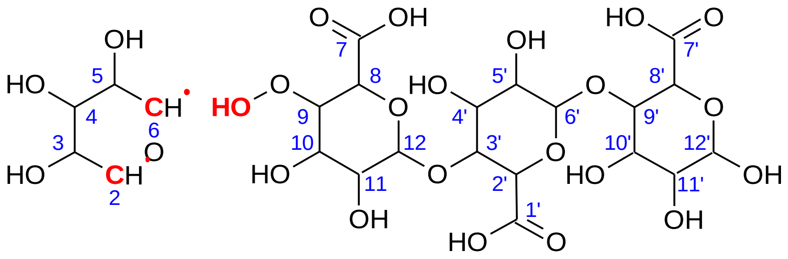	1.0

68	$C_{1'}OH$ and $C_{7'}OH$	1	H_2O formation, 2 CO_2 liberation, $C_{2'}=C_{3'}$ double bond formation, $C_{12}O$-$C_{3'}$ glycosidic bond cleavage, $C_{12}O^{\bullet}$ and $^{\bullet}C_{8'}$ formation<br><br>$C_{12}H_{17}O_{13}$ (369 Da) + $C_{10}H_{15}O_8$ (263 Da) + H_2O (18 Da) + $2CO_2$ (2·44 Da)	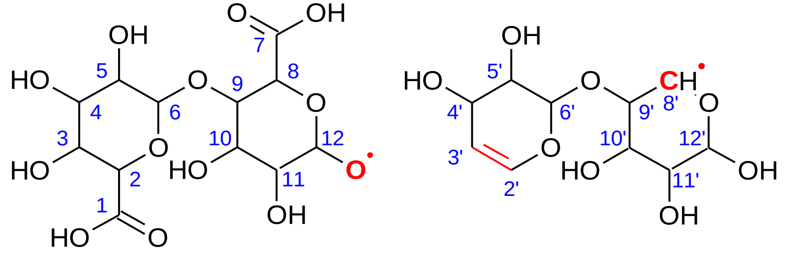	0.5
69	$C_{10}OH$ and later on C_1OH	1	C_9-C_{10} bond breaking (ring 2 opening and aldehyde formation), C_6-OC_9 glycosidic bond cleavage, H-abstraction from C_1OH (H_2O formation and CO_2 liberation), $^{\bullet}C_2$ and $^{\bullet}C_6$ formation<br><br>$C_{18}H_{24}O_{19}$ (544 Da) + $C_5H_8O_4$ (132 Da) + H_2O (18 Da) + CO_2 (44 Da)	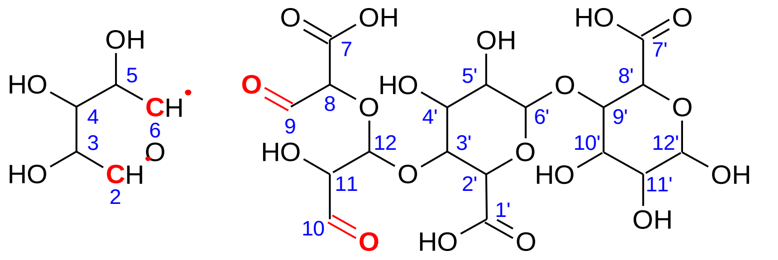	0.5
70	$C_{4'}OH$ and later on $C_{5'}OH$	1	$C_{3'}$-$C_{4'}$ bond breaking (ring 3 opening and aldehyde formation), H-abstraction from $C_{5'}OH$ (H_2O formation), C_{12}-$OC_{3'}$ glycosidic bond cleavage (aldehyde formation)<br><br>$C_{12}H_{15}O_{13}$ (367 Da) + $C_{12}H_{17}O_{12}$ (353 Da) + H_2O (18 Da)	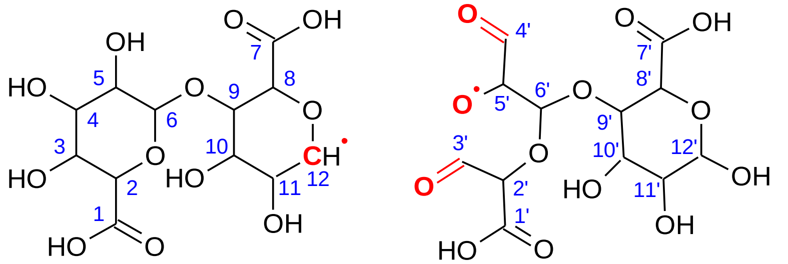	0.5
71	$C_{4'}OH$ and C_7OH	4	H-abs. from $C_{4'}OH$: $C_{3'}$-$C_{4'}$ bond breaking (ring 3 opening and aldehyde formation), H-abs. from C_7OH: CO_2 liberation, H_2O formation, C_8O-C_{12} bond breaking (ring 2 opening and aldehyde formation), $C_{2'}=C_{3'}$ bond formation, $C_{2'}$-$OC_{6'}$ and $C_{6'}$-$OC_{9'}$ bonds breaking, glycosidic bond cleavage<br><br>$C_{14}H_{19}O_{13}$ (395 Da) + $C_6H_9O_7$ (193 Da) + $C_3H_4O_3$ (88 Da) + H_2O (18 Da) + CO_2 (44 Da)	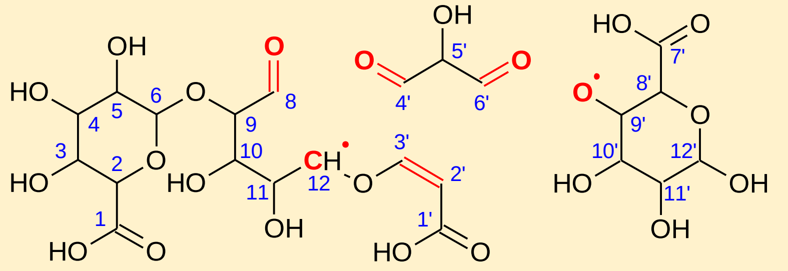	2.0

72	C_5OH and C_2H	1	H_2O formation, C_5-C_6 bond breaking (ring 1 opening and aldehyde formation), $^{\bullet}C_2$ and $^{\bullet}C_6$ formation<br><br>$C_{24}H_{32}O_{25}$ (720 Da) + H_2O (18 Da)	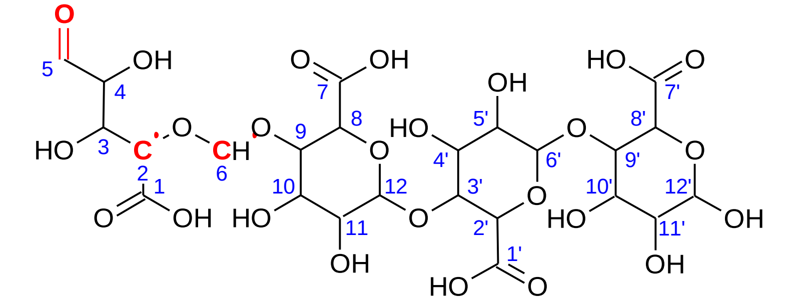	0.5
73	C_5OH and later on C_9H	1	C_5-C_6 bond breaking (ring 1 opening and aldehyde formation), H-abstraction from C_9H (H_2O formation), C_6O-C_9 glycosidic bond cleavage<br><br>$C_{18}H_{24}O_{18}$ (528 Da) + $C_6H_8O_7$ (192 Da) + H_2O (18 Da)	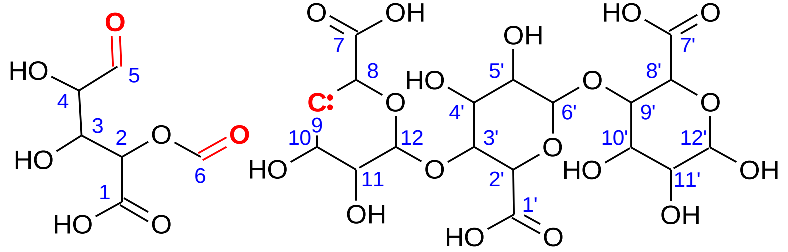	0.5
74	$C_{5'}OH$ and later on $C_{11'}OH$	1	$C_{5'}$-$C_{6'}$ bond breaking (ring 3 opening and aldehyde formation), H-abstraction from $C_{11'}OH$ (H_2O formation), $C_{6'}O$-$C_{9'}$ bond breaking, $C_{9'}$=$C_{10'}$ double bond formation and $C_{10'}$-$C_{11'}$ bond breaking (glycosidic bond cleavage, ring 4 opening and aldehyde formation)<br><br>$C_{18}H_{24}O_{19}$ (544 Da) + $C_6H_8O_6$ (176 Da) + H_2O (18 Da)	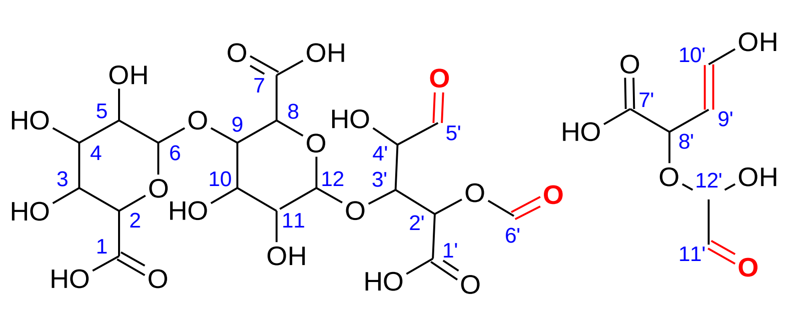	0.5
75	$C_{8'}H$	1	$C_{6'}O$-$C_{9'}$ glycosidic bond cleavage, $C_{8'}$=$C_{9'}$ formation, $C_{6'}O$-OH formation<br><br>$C_{18}H_{26}O_{20}$ (562 Da) + $C_6H_8O_6$ (176 Da)	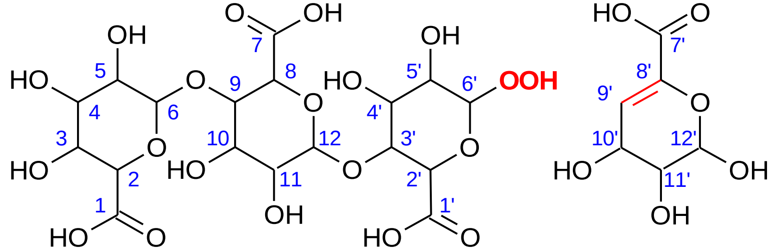	0.5

No.	Binding of O atom	Number of events	Description	Structure	%
76	C_6-**O**-C_9	1	Binding at C_6-**O**-C_9<br>$C_{24}H_{34}O_{26}$ (738 Da)	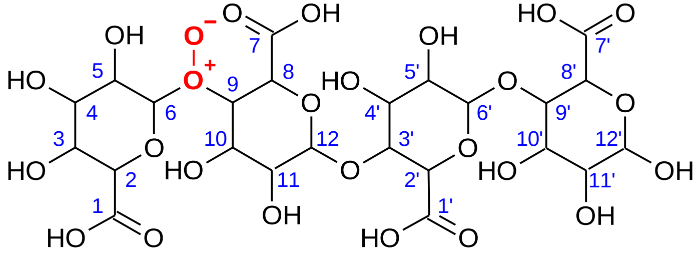	0.5
77	C_2-**O**-C_6	1	Binding at C_2-**O**-C_6<br>$C_{24}H_{34}O_{26}$ (738 Da)	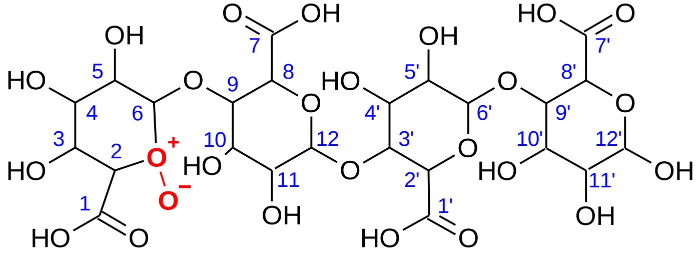	0.5
78	**C_1-C_2**	1	C_1-C_2 breaking, C_1-**O**-C_2 formation<br>$C_{24}H_{34}O_{26}$ (738 Da)	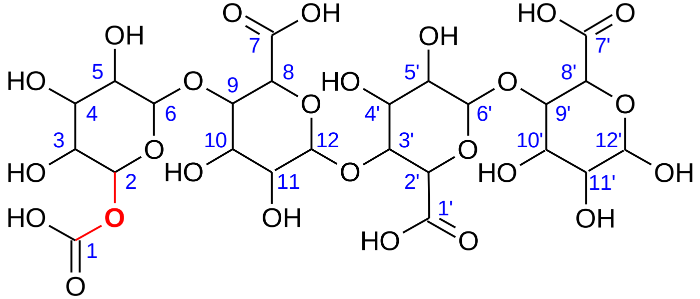	0.5
79	**$C_{1'}$-$C_{2'}$**	1	$C_{1'}$-$C_{2'}$ breaking, $C_{1'}$-**O**-$C_{2'}$ formation<br>$C_{24}H_{34}O_{26}$ (738 Da)	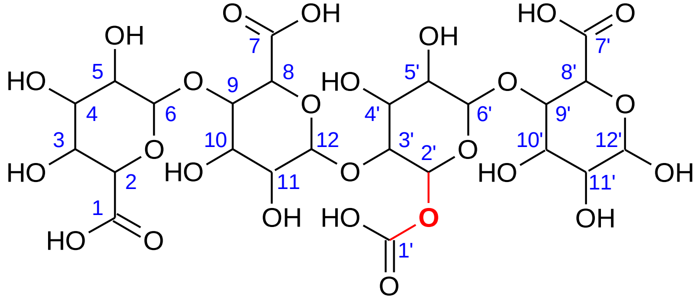	0.5

80	**$C_{7'}$-$C_{8'}$**	1	$C_{7'}$-$C_{8'}$ breaking, $C_{7'}$-**O**-$C_{8'}$ formation<br>$C_{24}H_{34}O_{26}$ (738 Da)	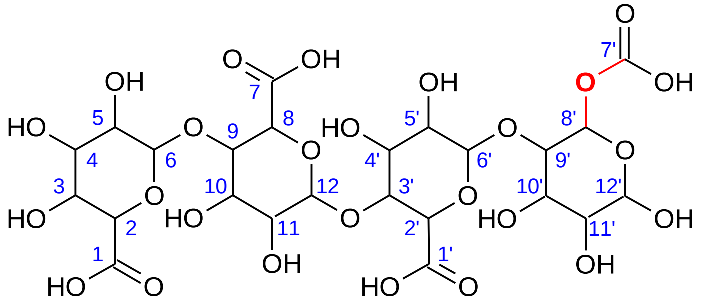	0.5
81	$C_{1'}$**O**	2	$C_{1'}$O-**O** formation<br>$C_{24}H_{34}O_{26}$ (738 Da)	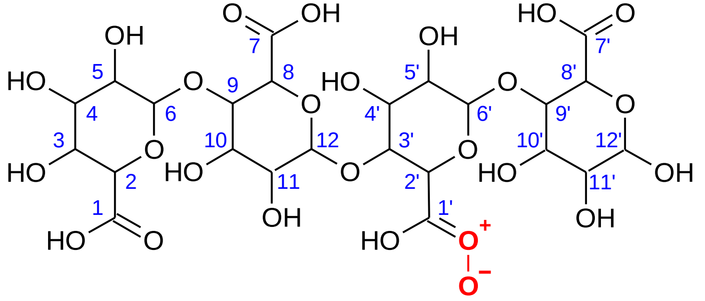	1.0
82	$C_{7'}$**O**	3	$C_{7'}$O-**O** formation<br>$C_{24}H_{34}O_{26}$ (738 Da)	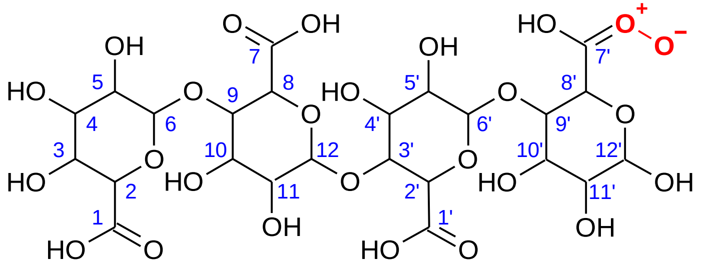	1.5
83	C_3**OH**	2	C_3O(-**O**)H formation<br>$C_{24}H_{34}O_{26}$ (738 Da)	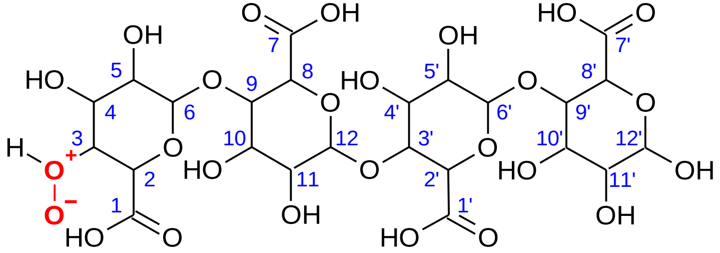	1.0

84	C_3**O**H	1	C_3O-**O**-C_1 formation, H-transfer from C_3OH to C_1O (diol formation), C_1-C_2 and C_1O-OC_3 bonds breaking (H_2CO_3 formation), C_3-C_4 bond breaking and C_2-C_4 bond formation (aldehyde and small ring formation)<br><br>$C_{23}H_{32}O_{23}$ (676 Da) + CH_2O_3 (62 Da)	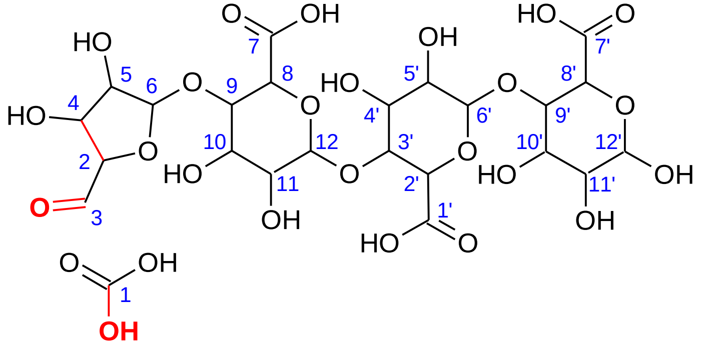	0.5
85	C_{10}**O**H	1	$C_{10}O$(-**O**)H formation<br><br>$C_{24}H_{34}O_{26}$ (738 Da)	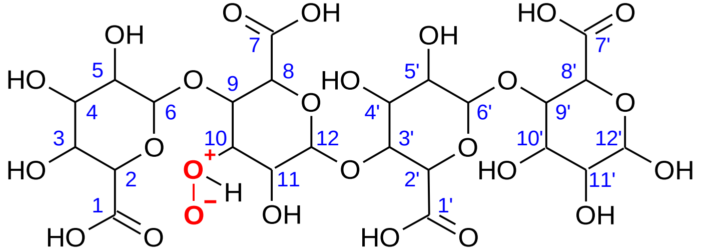	0.5
**Total**		**200**			**100**
