# Supplementary material for: Modeling Plasma-Induced Modifications in Alginate Biopolymers at the Atomic Scale
Source: J Phys Chem C Nanomater Interfaces. 2025 May 1;129(19):8927–36. doi: 10.1021/acs.jpcc.5c01565 (PMC12133024; doi:10.1021/acs.jpcc.5c01565)
Supplement: Supplementary file 2 [file jp5c01565_si_002.zip › DATA_Input_files/readme.docx]

**SUPPORTING INFORMATION**

**Modeling Plasma-Induced Modifications in Alginate Biopolymers at the Atomic Scale**

Maksudbek Yusupov^a,b,c,1,*^, Francesco Tampieri^d,e,f,1,*^, Shakhrizoda Matnazarova^g^, Nosir Matyakubov^h^, Cristina Canal^d,e,f^, Annemie Bogaerts^c^

^a^	Institute of Fundamental and Applied Research, National Research University TIIAME, Tashkent 100000, Uzbekistan

^b^	Department of Information Technologies, Tashkent International University of Education, Tashkent 100207, Uzbekistan

^c^	Research group PLASMANT, Department of Chemistry, University of Antwerp, Antwerp 2610, Belgium

^d^	Biomaterials, Biomechanics and Tissue Engineering Group, Department of Materials Science and Engineering and Institute for Research and Innovation in Health (IRIS), Universitat Politècnica de Catalunya - BarcelonaTech (UPC), Barcelona 08019, Spain

^e^	Barcelona Research Centre in Multiscale Science and Engineering (CCEM), UPC, Barcelona 08019, Spain

^f^	Centro de Investigación Biomédica en Red de Bioingeniería, Biomateriales y Nanomedicina (CIBER-BBN), Instituto de Salud Carlos III, Madrid 28029, Spain

^g^	Arifov Institute of Ion-Plasma and Laser Technologies, Academy of Sciences of Uzbekistan, Tashkent, Uzbekistan

^h^	Department of Physics, Urgench State University, Urgench 220100, Uzbekistan

*	Corresponding author.

^1^	Shared first author.

*e-mails:* [maksudbek.yusupov@uantwerpen.be](mailto:maksudbek.yusupov@uantwerpen.be) (M. Yusupov), [francesco.tampieri@upc.edu](mailto:francesco.tampieri@upc.edu) (F. Tampieri)

----------------------------------------------------------------------------------------------------------------------------------------

### ****Instructions for Running DFTB-MD Simulations for O Atom Impact on Alginate Tetramer:****

1. **Install DFTB+ Simulation Package**
   - Download and install the **DFTB+ simulation package** from the official website: <https://dftbplus.org/index.html>.
   - Follow the installation instructions provided on the website for your operating system.
2. **Download Parameter Set**
   - Obtain the **3ob-3-1 parameter set** from the DFTB website: <https://dftb.org/parameters.html>.
   - Ensure the parameter files are correctly placed in the directory specified in the DFTB+ input file.
3. **Energy Minimization Simulation**
   - Navigate to the **'Energy_minimization'** folder. This folder contains the necessary files to run the energy minimization simulation:
     - **dftb_in.hsd**: Input file for the DFTB+ simulation.
     - **struc.gen**: Structure file of the alginate tetramer.
     - **submit.sh**: Shell script to submit the job to the supercomputer cluster.
   - Run the simulation by executing the following command: sbatch submit.sh
4. **Equilibration Simulation**
   - Proceed to the **'Equilibration'** folder. This folder contains the following files:
     - **dftb_in.hsd**: Input file for the equilibration run.
     - **minimized.gen**: Structure file obtained from the **energy minimization** step.
     - **submit.sh**: Shell script to submit the equilibration job.
   - Perform **equilibration for 1.2 ns** by running: sbatch submit.sh
5. **Configuration Extraction**
   - After equilibration, extract **5 configurations of alginate** from the **last 50 ps** of the run using the **geom.xyz** file.
   - These configurations will be used for subsequent **O atom impact simulations**.
6. **Preparation of Initial Geometries**
   - For each of the **5 extracted configurations**, generate **20 geometry files** by randomly placing a **single O atom** around the alginate tetramer.
   - In the **'MD_100runs'** folder:
     - There are **5 directories**, each containing **20 run subfolders**, resulting in **100 runs** in total.
7. **Particle Impact Simulations**
   - In the **'MD_100runs'** folder:
     - The folder contains the input file **dftb_in.hsd**, **5 structure directories** (each with 20 subfolders), and the following shell scripts:
       - **operation1.sh**: Copies all necessary files to each run subfolder (100 in total).
       - **operation2.sh**: Submits each job in each run folder to the supercomputer cluster.
       - **submit.sh**: General job submission script.
   - Perform the simulations by running the following commands sequentially: “bash operation1.sh” and “bash operation2.sh”
8. **Additional Particle Impact Simulations**
   - Repeat the process by creating a new **'MD_100runs'** folder.
   - Perform **another set of 100 particle impact simulations**, bringing the total to **200 MD runs**.
